# Integrated Assessment of Benthic Bacterial Community Physiology, Structure, and Function Across C, N, P, and S Gradients in Lake Villarrica Sediments, Chile

**DOI:** 10.3390/microorganisms13112544

**Published:** 2025-11-07

**Authors:** Tay Ruiz-Gil, Sebastián Elgueta, Giovanni Larama, Joaquín-Ignacio Rilling, Anthony Hollenback, Deb P. Jaisi, Diego Valdebenito, Bryan M. Spears, Marco A. Campos

**Affiliations:** 1 Laboratorio de Investigación Interdisciplinaria en Microbiología Aplicada (LIMA), Centro de Investigación en Ciencias de la Salud, Departamento de Procesos Diagnósticos y Evaluación, Facultad de Ciencias de la Salud, Universidad Católica de Temuco, Temuco 4810399, Chile; tayruizbio@gmail.com (T.R.-G.); 2 Escuela de Nutrición y Dietética, Facultad de Ciencias de la Rehabilitación y Calidad de Vida, Universidad San Sebastián, Sede Los Leones, Providencia, Santiago 7500000, Chile; sebastian.elgueta@uss.cl; 3 Biocontrol Research Laboratory, Universidad de La Frontera, Temuco 4780000, Chile; giovanni@larama.cl; 4 Millennium Institute Center for Genome Regulation (MI-CGR), Santiago 8331150, Chile; ignacio.rilling@gmail.com; 5 Department of Plant and Soil Sciences, University of Delaware, Newark, DE 19716, USA; tonyh@udel.edu (A.H.); jaisi@udel.edu (D.P.J.); 6 UK Centre for Ecology & Hydrology, Bush Estate, Penicuik, Midlothian, Edinburgh EH26 0QB, UK; spear@ceh.ac.uk

**Keywords:** physiology, diversity, function, eutrophication, bed sediments, biogeochemistry

## Abstract

Benthic bacterial communities play a critical role in nutrient cycling and are highly sensitive to environmental pollution. This study aimed to investigate the physiological, compositional and functional responses of bacterial communities across a range of carbon (C), nitrogen (N), phosphorus (P), and sulfur (S) gradients in sediments from Lake Villarrica, Chile. Sediment samples were collected from 5 sites representing a gradient of nutrient pressure from the lake basin (NL < PuB < PoP < SL < VB). Nutrient forms (TC, TN, TP, TS, and OM) were chemically quantified. Community function was assessed via community-level physiological profiles (CLPPs) using Biolog^®^ EcoPlates (C substrates), PM3B (N substrates), and PM4A (P and S substrates). Function and composition were assessed based on total bacterial and functional nutrient-cycling gene abundances (16Sr RNA, *chi*A, *mcr*A, *nif*H, *amo*A, *nos*Z, *pho*D, *pqq*C, *sox*B, *dsr*A) using qPCR and 16S rRNA metabarcoding, respectively. In general, the CLPPs were higher for C substrates, followed by P, S, and N substrates, with metabolism of organic forms of these nutrients preferential, and P-cycling genes were the most abundant in the lake. Spatially, the most nutrient-enriched site (VB) showed a significantly (*p* ≤ 0.05) higher nutrient content (e.g., 5.4% TC, 0.54% TN, 1302.8 mg kg^−1^ TP and 854.1 mg kg^−1^ TS) and total bacterial abundance (2.9 × 10^11^ gene copy g^−1^ dw sediment) but displayed lower CLPPs (from 0.63 to 1.02 AWCD) and nutrient-cycling gene abundances (e.g., 9.1 × 10^1^, 2.7 × 10^3^, 3.6 × 10^3^ and 4.7 × 10^3^ gene copy g^−1^ dw sediment for *chia*A, *nif*H, *pho*D and *dsr*A, respectively) compared to the less nutrient-enriched sites (e.g., NL). The bacterial community composition shifted accordingly, with Bacillota enriched in VB and Planctomycetota occurring more frequently in less nutrient-exposed sites. Functional prediction analysis revealed enhanced methanotrophy and sulfate respiration in nutrient-rich sediments, whereas nitrification and organic P (P_o_) mineralization dominated in less impacted areas. The results demonstrate that nutrient enrichment constrains bacterial functional diversity in Lake Villarrica and, so, may be useful indicators of environmental stress to be considered in pollution monitoring programmes.

## 1. Introduction

Carbon (C), nitrogen (N), phosphorus (P), and sulfur (S) are key biogenic elements that drive primary production and biogeochemical cycles in lakes [1,2]. They support microbial autotrophy and heterotrophy, promoting biomass growth and replication [3,4,5]. Their elemental balance, also known as nutrient stoichiometry, is crucial control for shaping freshwater communities and their interactions [3,6]. However, anthropogenic pollution leading to changes in nutrient loading (i.e., eutrophication) to lakes may disrupt this balance, leading to increased biomass accrual and enrichment of lake-bed sediments with organic matter (OM) [5,7,8]. The aerobic decomposition of OM by the microbial community consumes oxygen as an electron donor and plays a key role in the REDOX processes of lake-bed sediments [9]. When OM loading is high, elevated biological oxygen demand may deplete the oxygen supply, increasing the likelihood of hypoxia, a condition commonly reported across eutrophic lakes. Nutrient enrichment, derived primarily from the loss from agricultural lands, may also alter the supply of key electron donors in the REDOX series (e.g., NO_3_^−^ from agriculture and wastewater) [10]. Under these conditions, the REDOX sequence progresses as electron donors are depleted, altering microbial community structure and function through interconnected processes that ultimately shape nutrient stoichiometry in the overlying water. Hypoxia has been reported across many of the world’s large lakes, including Lakes Erie, Mendota, and Okeechobee in the United States [11,12,13], Lake Winnipeg in Canada [14], and Lake Taihu in China [8]. However, responses in benthic microbial community structure and function in large lakes across environmental gradients remain poorly understood compared with those of pelagic communities. Geographically, studies of this kind focusing on lakes in the southern cone of Latin America are particularly sparse [15].

Sediments are hotspots for coupled transformations of nutrients via electron transport reactions, driven particularly by benthic bacterial communities [9]. Organic matter decomposition generates carbon dioxide (CO_2_) [16] and approximately 16% of global methane (CH_4_) emissions [3]. This process requires the supply of nitrate (NO_3_^−^) and nitrite (NO_2_^−^), which influence heterotrophic denitrification, and the production rates of nitrous oxide (N_2_O) [10]. Additionally, heterotrophic denitrification is influenced by sulfide (S^2−^) availability and the presence of sulfide-oxidizing bacteria (SOB) that utilize S^2−^ as an energy source and NO_3_^−^ as an electron acceptor [17,18]. At the same time, under anoxic conditions, the reduction of sulfate (SO_4_^2−^) into S^2−^ by sulfur-reducing bacteria (SRB) favors the precipitation of sulfide minerals such as FeS_2_, promoting the solubilization of P originally bound to Fe oxide minerals [14,19]. While many coupled reactions indirectly mobilize P, bacteria can directly solubilize inorganic P (P_i_) or mineralize organic P (P_o_) to use Pi, along with C, N, or S to fuel the enzymatic reactions [5,20].

Nutrient flux between lake-bed sediments and the overlying waters controls lake water quality. Yet, the role of the microbial community in driving such processes remains a key knowledge gap in lake and reservoir systems [15]. Nonetheless, recent methodological advances allow applying genomic and physiological functions of benthic bacterial communities and provide valuable insights into their roles in lake nutrient dynamics [4]. By assessing the abundance and correspondence of functional traits in nutrient transformation processes by using enzyme-coding genes, it is now possible to map bacterial metabolism in the context of nutrient cycling at the community and ecosystem scales [21]. Moreover, analyzing the physiological diversity of bacterial communities, which refers to their ability to metabolize nutrients and other natural or xenobiotic molecules [22], can provide valuable insights into ecological stress across environmental gradients [23], including nutrient enrichment. It means approaches such as community-level physiological profiling (CLPP) provide a standardized assessment of community functional responses to nutrient pollution [23,24].

Lake Villarrica (39°15′00″ S, 72°02′00″ W; 214 m.a.s.l.) is a glaciogenic lake situated at the western foothills of the Chilean Andes and is a key tourist hotspot and related economic activities [25,26]. Over the past five decades, significant changes in land use within its watershed have adversely impacted the lake’s water quality through accelerated nutrient enrichment and frequent occurrences of eutrophication [26]. Recognizing the ecological significance of this lake, the Chilean Ministry of Environment implemented a secondary environmental quality standard to manage land sources and reduce the nutrient pollution [27]. As is the case in many such analogous lakes globally, this plan was fundamentally based on estimates of catchment nutrient loads and tracking classical physicochemical and biochemical water quality indicators. Recent studies have revealed correlations among the frequency of toxigenic cyanobacterial blooms [28,29], plankton abundance [26,30], and abundances of N and P-cycling genes in the bacterial community [31], suggesting that benthic processes may be a more important driver of water quality than originally thought. Here, we took an in-depth study in five sites under national monitoring program but with contrasting catchment nutrient loads to (i) assess nutrient status in the sediment beds underlying the monitoring sites, (ii) conduct an detailed evaluation of indicators of bacterial community specific physiological, structural, and functional diversity, and (iii) identify physico-chemical and microbial associations including sediment nutrient content and bacterial community indicators. Outcomes of this study are expected to provide insights into the role the coupled factors in driving nutrient enrichment in Lake Villarrica. Given the paucity of previous studies in this region, we compare our results with similar studies to inform interpretation.

## 2. Materials and Methods

### 2.1. Lake Description

**Figure 1:**
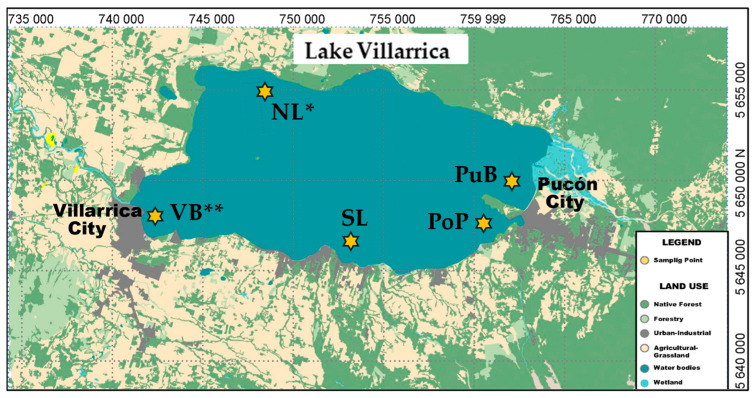
Location of sampling sites in Lake Villarrica (39°15′00″ S 72°02′00″ W), which were coded as Pucón City Bay (PuB), Poza Port (PoP), South Lake (SL), North Lake (NL), and Villarrica City Bay (VB). The single asterisk (*) denotes the site situated in the basin of low anthropogenic impact, historically used as a reference site, and the double asterisk (**) denotes the site with the expected highest anthropogenic impact. PuB, PoP, and SL are considered intermediate-impacted sites. The land-use data also indicate that VB is exposed to a catchment with higher-intensity land use, in contrast to NL.

The study was spread across five sampling sites of the Lake Villarrica basin, including the following areas: North Lake (NL), Pucón City Bay (PuB), Poza Port (PoP), South Lake (SL), and Villarrica City Bay (VB) (Figure 1). These five sites are a part of the national monitoring network of inland waters established by the Chilean Ministry of Environment in 2016 [32]. On average, the lake receives 3.06 × 10^−2^ Tg year^−1^ of total P (TP) and 1.48 × 10^−3^ Tg year^−1^ of total N (TN) from diffuse and point sources, primarily located along the shoreline between Pucon and Villarrica cities, where the PuB, PoP, SL, and VB sampling sites are located [33]. The diffuse sources, such as different land uses including agriculture, forestry, and livestock and lake-edge homes, contribute close to 1.82 × 10^−2^ Tg year^−1^ of TP and 7.11 × 10^−2^ Tg year^−1^ of TN. In contrast, point sources such as wastewater discharges from near settlements and aquaculture contribute 1.24 × 10^−2^ Tg year^−1^ of TP and 7.66 × 10^−2^ Tg year^−1^ of TN [33]. In general, all these sources are denser near SL and VB sites, with the VB site being classified as the most nutrient-impacted according to the national monitoring [32,33]. These sources have lower inputs to PuB and PoP, with NL being considered a ‘reference site’ due to the low inputs from anthropogenic nutrient sources in its sub-watershed. However, during summer, the Puelche wind, a type of Foehn wind typically found in southern Chile, frequently drags superficial bed sediment from the south lakeside to the north lakeside, changing some properties of the NL site [31]. In summary, we consider the sites to be exposed to different levels of anthropogenic nutrient inputs form their sub-watersheds in the order: NL < PuB < PoP < SL < VB. Standardized (z-score) plots illustrating this series are provided in Supplementary Materials Figure S1.

### 2.2. Sediment Sampling and Characterization of In-Situ Physicochemical Properties

Sediment sampling was conducted at each site during summer 2023 using a Petersen-type grab sampler, following the procedure described by Campos et al. [34]. Four independent replicate sediments were collected at each site below the depths of 20 to 30 m. Each grab sample retrieved included a surface sediment layer of less than 10 cm thickness, corresponding to the active microbial interface. Replicates were taken approximately 5 m apart to account for spatial heterogeneity of each site. Each sample was measured for in-situ pH, temperature (Temp), electrical conductivity (EC), dissolved oxygen (DO), and redox potential (ORP) with a multiparameter probe (YSI ProDSS, Yellow Springs Instrument, OH, USA; Table 1). Then, approximately 800 g of sediment was aseptically placed into sterile plastic bags, kept at 4 °C, and then transported to the Interdisciplinary Research Laboratory in Applied Microbiology (LIMA) at the Universidad Católica de Temuco (UCT). At LIMA, each sample was split into different aliquots as follows. One aliquot set was for CLPP analysis (stored at 4 °C), another for nutrient analyses (freeze-dried), and the third for the genomic DNA (gDNA), nutrient-cycling gene abundances, and bacterial community analyses (stored at −80 °C).

### 2.3. Nutrient Content of Sediments

Chemical analyses were conducted on the freeze-dried sediment samples to determine the content of total carbon (TC), total organic carbon (TOC), organic matter (OM), total nitrogen (TN), total phosphorus (TP), and total sulfur (TS) (Table 1). TC and TN were directly measured by combustion using an Elementar VarioMax CN Cube Analyzer (Elementar Americas, Mt. Holly, NJ, USA). For TOC estimation, samples were acidified with HCl to eliminate carbonates and then dried and combusted, whereas OM was determined by loss on ignition (550 °C for 4 h). Both TOC and OM were expressed as a percentage of dry weight (dw) [35,36]. TP and TS were analyzed via inductively coupled plasma optical emission spectroscopy (ICP-OES) after microwave-assisted acid digestion in a CEM MARS6 microwave digestion system (CEM, Matthews, NC, USA) following the EPA Method 3051A for analysis of sediments [37].

### 2.4. Community-Level Physiological Profile (CLPP) of Bacterial Communities

Sediment samples were evaluated to determine the capability of bacterial communities to metabolize from 31 to 95 different C, N, P, and S substrates, using Biolog^®^ EcoPlates™ (31 C substrates), PM3B (95 N substrates), and PM4A (divided into 59 P and 35 S substrates) microwell plates (Biolog Inc., Hayward, CA, USA) using colorimetric methods. Briefly, triplicate fresh sediment sub-samples were independently diluted (1:100) into four sterilized modified M9 media without C (CM9), N (NM9), P (PM9), or S (SM9) sources. For NM9, PM9, and SM9 media (Supplementary Materials Table S2), REDOX dye triphenyltetrazolium chloride (TTC; Biolog Redox Dye Mix MA, Biolog Inc., Hayward, CA, USA) was added at a concentration of 0.01% (*w*/*v*), whereas for EcoPlates™, the dye was already included. Triplicate treatments of different nutrient sources and controls were used.

Each well was inoculated under sterile conditions with 12.5 µL of the corresponding sediment dilution, where single sources of C, N, P, or S were provided in the microwells. The plates were immediately sealed to prevent external contamination and dehydration, then placed in a shaker incubator at 25 °C in the dark. Absorbance readings at 590 nm were taken at 0, 6, 12, 24, 36, 48, 60, 72, and 96 h using a Multiskan™ GO Microplate Spectrophotometer (Thermo Fisher Scientific, Hercules, CA, USA). To correct for the sediment suspension effect, the average absorbance of the control wells was subtracted from the absorbance of each substrate well at every time point.

The corrected absorbance data were used to calculate the Average Well Color Development (AWCD) as described by Sofo and Ricciuti [22]. Given the wide range of nutrient sources, the averaged AWCD for each sediment sample at each time point during the incubation period was recorded and used to evaluate growth kinetics as well as to calculate the area under the curve (AUC) (Tables S3 and S4). Additionally, a heatmap was constructed based on separate AWCDs for nutrient functions and their molecular structures in each microwell plate. The classification of C sources into functional categories of amino acids, amines, glucides, carboxylic acids, phenolic compounds, and polymers was conducted as recommended by Garland and Mills [38]. However, the classification into functional categories of (i) N sources from the PM3B (ammonia, nitrite, nitrate, amino acids and derivatives, amines and derivatives, amides and derivatives, nucleobases and derivatives, and peptides), (ii) P sources from the PM4A (phosphate, polyphosphate, hypo and thiophosphate, phosphomonoesters, phosphodiesters, phosphotriesters, phosphonates, inositol phosphate, and other P_o_ compounds) and (iii) S sources from the PM4A (sulfates, thiosulfates, tetrathionates, thio and dithiophosphates, S-amino acids and derivatives, and other S_o_ compounds). Individual absorbance for each C, N, P, and S substrate at the last time point of the kinetic growth is provided in Supplementary Materials Table S5.

### 2.5. Genomic DNA Extraction

gDNA extraction was carried out using the DNeasy^®^ PowerSoil^®^ kit (Qiagen Inc., Germantown, MD, USA). First, 500 mg of sediment was transferred to the kit bead tubes and exposed to three rounds of 15 min freeze–thaw cycles (−80 °C/65 °C) to improve DNA yields, prior to homogenization. The remaining steps were performed according to the manufacturer’s instructions, and 50 µL of gDNA were eluted and stored at −20 °C for analysis. The quality and quantity of gDNA were measured with a QuantiFluor ONE-DNA Kit on a Quantus fluorimeter (Promega, Madison, WI, USA). The gDNA was later used to analyze the total bacterial and specific functional-nutrient gene abundances, as well as the bacterial community composition, predicted functionality, and associations with nutrient content and specific functional cycling gene abundances.

### 2.6. Absolute Quantification of Total Bacterial Community and Nutrient-Cycling Functional Genes

The absolute abundance of total bacterial (16S rRNA gen) community and the occurrence and abundance of bacterial functional enzyme-encoding genes involved in key steps of the C (chitinase group A [*chia*A] and methyl-coenzyme M reductase subunit alpha [*mcr*A]), N (dinitrogenase reductase [*nif*H], ammonia monooxygenase subunit A [*amo*A] and nitrous oxide reductase [*nos*Z]), P (alkaline phosphomonoesterase D [*pho*D] and pyrroloquinoline quinone synthase C [*pqq*C]), and S (sulfohydrolase [*sox*B] and dissimilatory sulfite reductase subunit A [*dsr*A]) cycling were determined using qPCR and a QuantStudio1™ Real-Time PCR System (ThermoFisher Scientific, Inc., Waltham, MA, USA). The qPCR reactions were performed using 5x HOT FIREPol^®^ EvaGreen^®^ qPCR Mix Plus (ROX) (Solis Biodyne, Tartu, Estonia) and ~25 ng μL^−1^ of DNA in 10 μL of reaction. The primer sets and conditions used for the quantification of the 16S rRNA [39], *chia*A [40], *mcr*A [41], *nif*H [42], *amo*A [43], *nos*Z [44], *pho*D [45], *pqq*C [46], *sox*B [47], and *dsr*A [48] bacterial genes are detailed in Supplementary Materials Table S6. The copy number estimation and standard curves used for absolute gene quantification were designed following the method described by Campos et al. [34].

### 2.7. Assessment of Bacterial Community Composition, Nutrient Content, and Functional Gene Abundance

Bacterial community composition, expected functionality, and associations with nutrient content and nutrient-cycling gene abundances were assessed through 16S rRNA gene metabarcoding sequencing and bioinformatic analyses. Briefly, the 16S rRNA gene libraries were subjected to PCR for 16S rRNA gene amplification using primers 341f and 805r [49], indexed according to the Illumina Nextera XT v2 kit (Illumina Co., San Diego, CA, USA), and pair-end sequenced on an Illumina MiSeq sequencer (Illumina Co.). The resulting sequences were analyzed using QIIME2 (https://qiime2.org/) and free R packages (version 4.0.2). The low-quality regions were trimmed to maintain an average quality score above 30. The alignments were based on SILVA version 138.1 [50]. The reads were rarefied to 92,178, and the chloroplast and mitochondrial sequences were removed. Differences among communities from each site for richness, alpha (Observed ASVs, Shannon, Pielou’s Evenness, and Faith’s Phylogenetic indices; Supplementary Materials Table S7) and beta diversity (principal coordinate analysis [PCoA]; Supplementary Materials Figure S3) indices were obtained and tested for significance by the Kruskal−Wallis test and PERMANOVA based on Bray−Curtis distances. The relative abundances at the phylum and family levels, and the intersecting Amplicon Sequence Variants (ASVs), were calculated and plotted for samples using the R project (https://www.r-project.org/) packages “ggplot2” and “UpSetR”.

To predict metabolic or other ecologically relevant functions of the bacterial communities, the Functional Annotation of Prokaryotic Taxa (FAPROTAX) database was used as described by Louca et al. [51]. The metagenome function of *chia*A, *mcr*A, *nif*H, *amo*A, *nos*Z, *pho*D, *pqq*C, *sox*B, and *dsr*A genes was predicted using the Phylogenetic Investigation of Communities by Reconstruction of Unobserved States (PICRUSt2) analyses [52], followed by a DESeq2 analysis for a differential contrast of the gene expression and abundances among samples [53]. Finally, the relationships among nutrient composition, corresponding specific functional nutrient cycling genes, and bacterial abundance at the family level were assessed using redundancy analysis (RDA) with Hellinger distances, as implemented in the “Vegan” package in R (Supplementary Materials Table S9).

The raw sequencing data generated in this study from DNA metabarcoding analysis were deposited in the Sequence Read Archive (SRA; https://www.ncbi.nlm.nih.gov/sra accessed on 11 April 2025) a of the National Center for Biotechnology Information (NCBI) under accession number PRJNA1356897.

### 2.8. Statistical Analysis

The data obtained from in-situ analysis, nutrient quantification, CLPP, and absolute abundance of total bacterial communities and functional nutrient-cycling genes in sediment samples were contrasted using one-way ANOVA with Tukey’s honest significant difference (HSD) test. All tests were performed with a 5% significance level (*p* ≤ 0.05), and values are shown as the means of three or four replicates and are presented as means ± standard deviations. Spearman’s rank correlation (ρ) was computed between qPCR-measured and PICRUSt2-predicted gene abundances across sites to evaluate monotonic concordance between observed and inferred functions (Supplementary Materials Table S8).

## 3. Results

### 3.1. Physicochemical Parameters and Nutrient Contents

The spatial comparison of the in-situ analysis (Table 1) revealed slight differences in sediment composition across the sample sites. In general, pH values were close to neutral (6.7 to 7.4), temperature ranged from 17.5 to 20.7 °C, EC ranged from 57.4 to 61.3 µS cm^−1^, and ORP ranged from −141.3 to −86.9 mV. However, significant differences (*p* ≤ 0.05) were observed in nutrient content (Table 1). Higher contents of TC (5.4 ± 0.1%), TOC (5.3 ± 0.2%), OM (14 ± 2.3%), TN (0.54 ± 0.01%), TP (1302.8 ± 12.5 mg kg^−1^), and TS (854.1 ± 15.1 mg kg^−1^) were reported for the VB site compared to the NL, PuB, PoP, and SL sites. In general, sediment nutrient content decreased along the following series: VB > NL > PuB | PoP | SL. Standardized (z-score) plots illustrating this gradient are provided in Supplementary Materials Figure S1.

### 3.2. Community-Level Physiological Profile (CLPP) Responses During Sediment Incubations

**Figure 2:**
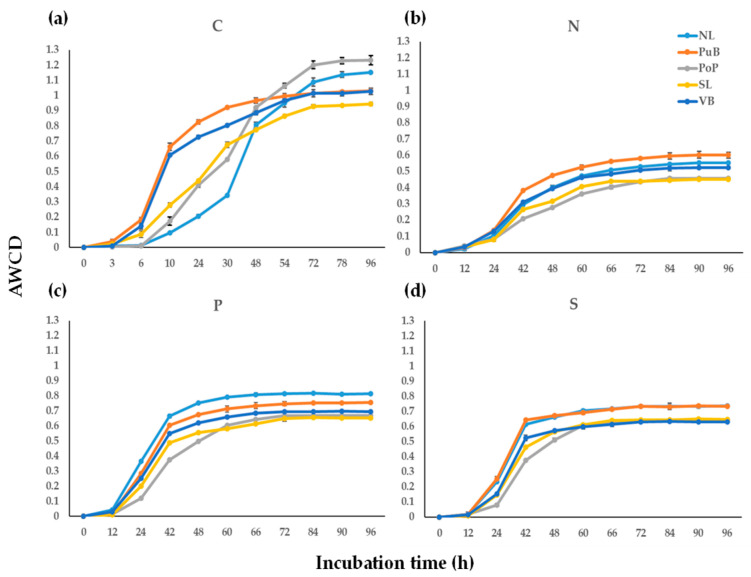
Average Well Color Development (AWCD) for (**a**) Biolog^®^ EcoPlates™ with 31 C substrates, (**b**) PM3B with 95 N substrates), (**c**) PM4A with 59 P and (**d**) with 35 S substrates incubated at 25 °C in the dark. Absorbances were corrected by subtracting control data at the endpoint AWCD (96 h). Values represent the mean (*n* = 3) ± standard deviation. Corresponding AUC values per site (mean ± 95% CI) are provided in Supplementary Materials Table S4.

**Figure 3:**
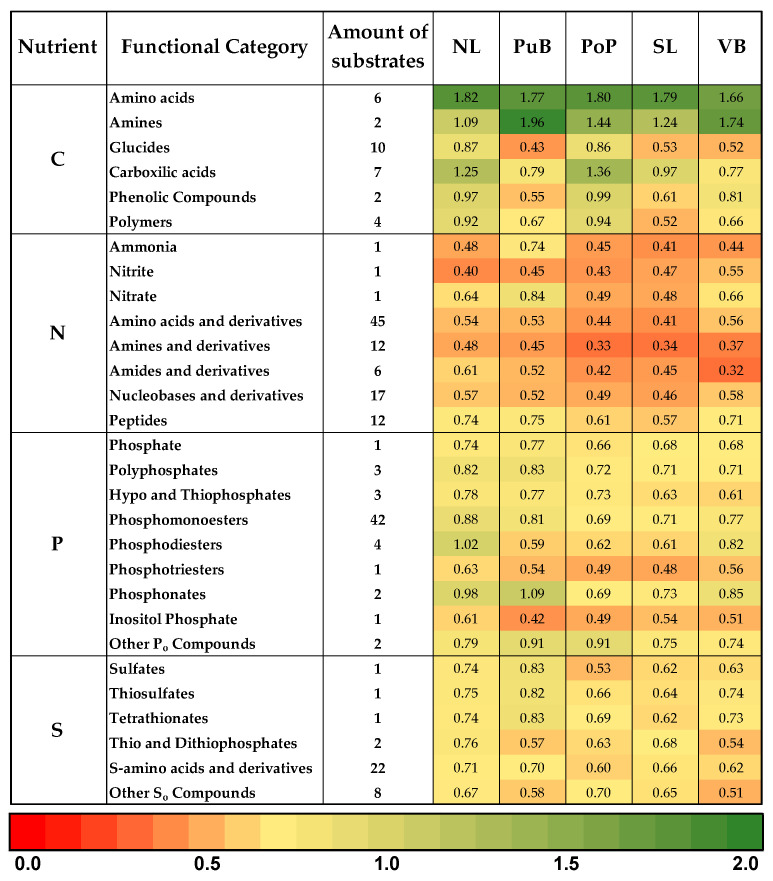
Heatmap of AWCD (from 0 to 2.0 units of absorbance) for the disaggregated nutrient categories for each of C, S, N, and P. Absorbances were measured for treatments and corrected against controls over the 96-h incubation period over 96 h. Values represent the mean (*n* = 3) replicates. A substrate layout and functional category mapping is provided as Supplementary Materials Figure S2.

The average AWCD in single kinetics, grouped for all C, N, P, and S substrates provided in the Biolog^®^ microwell plates, during the incubation period are shown in Figure 2). Further, AWCD at the end of the incubation for disaggregated functional nutrient categories for C, N, P and S substrates are shown in Figure 3. In general terms, the CLPP for all microwell plates initially showed significant statistical (*p* ≤ 0.05) differences from 6 h of incubation before stabilizing after 72 to 96 h. The AUC values (Supplementary Materials Table S4) confirmed site-specific differences in metabolic dynamics. Although VB showed relatively high AUC due to early color development, its final AWCD remained the lowest among sites, indicating an early but limited overall metabolic response (Supplementary Materials Table S3). The site VB had significantly lower AWCD values (*p* ≤ 0.05) across all microwell plates compared with all other sites. Among different nutrient sources, the AWCD for C sources (Figure 2a) showed the PoP site (1.23 ± 0.03) was significantly higher than those at NL, PuB, VB, and SL sites (from 0.94 ± 0.01 to 1.15 ± 0.01). In the case of N sources (Figure 2b), values of AWCD were significantly higher for the PuB site (0.60 ± 0.02) than for the NL, PoP, SL, and VB sites (from 0.45 ± 0.02 to 0.55 ± 0.01). For the P sources (Figure 2c), the peak AWCD was present in NL site (0.81 ± 0.03), followed by PuB, VB, PoP, and SL sites (from 0.65 ± 0.01 to 0.76 ± 0.01). Similarly, for the S sources (Figure 2d), NL (0.74 ± 0.03) and PuB (0.74 ± 0.02) sites were the most active, followed by PoP, SL, and VB sites (from 0.63 ± 0.02 to 0.65 ± 0.02). Comparing among all sites, the CLPP appeared to be most active in metabolizing C (0.94 ± 0.0 to 1.23 ± 0.03), followed by P (AWCDs from 0.67 ± 0.01 to 0.81 ± 0.03), S (from 0.63 ± 0.02 to 0.74 ± 0.02), and N (from 0.45 ± 0.02 to 0.60 ± 0.02) sources.

Analyzing the preference of C, N, P, and S compounds (Supplementary Materials Table S5), the heatmap (Figure 3) confirmed that bacterial communities were more active when utilizing C sources, followed by P, S, and N. From the C sources, amino acids (1.77 ± 0.07), followed by amines (1.49 ± 0.36) and carboxylic acids (1.03 ± 0.26), elicited the greatest response across all sample sites. These groups were more metabolized in NL, PuB, PoP, and SL (ranging from 0.97 to 1.82) than in VB (ranging from 0.77 to 1.66) site. In the case of P sources, sediments showed a greater inclination to metabolize phosphonates (0.87 ± 0.17), other P_o_ compounds (0.82 ± 0.09), phosphomonoesters (0.77 ± 0.08), and polyphosphates (0.76 ± 0.06). In this case, NL, PuB, and PoP (ranging from 0.69 to 1.09) sites were more active than SL and VB (ranging from 0.68 to 0.85). Regarding S sources, a greater inclination for thiosulfates (0.72 ± 0.07) and tetrathionates (0.72 ± 0.08) was observed. Differently, NL, PuB, and VB (from 0.73 to 0.82) were more active than PoP and SL (from 0.62 to 0.69). Finally, N sources prioritized the utilization of peptides (0.68 ± 0.08), nitrate (0.62 ± 0.15), and nucleobases and derivatives (0.52 ± 0.05). The PuB, NL, and VB (from 0.52 to 0.84) were higher than PoP and SL (0.46 to 0.61) in use these N sources.

### 3.3. Variation of Total Bacterial Community and Functional Nutrient-Cycling Genes

Our analysis of total bacterial abundance and functional genes for C, N, P, and S cycling (Table 2) confirmed statistically significant differences (*p* ≤ 0.05) among the sample sites. Overall total bacterial abundance was highest in the VB site (2.9 × 10^11^ ± 6 × 10^10^ gene copy g^−1^), followed by PoP, SL, NL, and PuB (from 7.4 × 10^9^ ± 6 × 10^6^ to 1.5 × 10^11^ ± 8 × 10^10^ gene copy g^−1^). However, across sites, where total bacterial abundance was highest, nutrient-cycling gene abundance was lowest. For example, NL had higher abundances of *chi*A, *amo*A, and *nos*Z (from 8.5 × 10^1^ ± 2 × 10^1^ to 2.4 × 10^4^ ± 4 × 10^3^ gene copy g^−1^) than PuB, PoP, SL, and VB sites (from 4.0 × 10^0^ ± 2 × 10^1^ to 6.3 × 10^3^ ± 8 × 10^1^ gene copy g^−1^). The abundance of *chia*A was lowest in the VB site (9.1 × 10^1^ ± 2 × 10^1^ gene copy g^−1^). Abundances of *nif*H, *pho*D, and *sox*B genes were similar for NL, PoP, and SL sites (from 6.0 × 10^3^ ± 2 × 10^3^ to 2.6 × 10^5^ ± 6 × 10^4^ gene copy g^−1^), which were significantly higher than VB (from 1.5 × 10^2^ ± 6 × 10^1^ to 3.6 × 10^3^ ± 2 × 10^3^ gene copy g^−1^). In contrast, the *mcr*A gene was significantly higher in NL and VB sites (from 2.1 × 10^3^ ± 8 × 10^2^ to 3.8 × 10^3^ ± 1 × 10^3^ gene copy g^−1^) than PoP, PuB, and SL (from 5.8 × 10^2^ ± 5 × 10^1^ to 8.0 × 10^2^ ± 1 × 10^2^ gene copy g^−1^). For *pqq*C, no significant differences were observed among sites.

### 3.4. Changes in Bacterial Community Composition and Functionality, and the Relationship of Nutrient Content with Specific Functional-Cycling-Gene Abundances Among Sites

No statistically significant differences were observed between the sites for bacterial community richness using Faith’s Phylogenetic Diversity Index (Supplementary Materials Table S7). However, Shannon and Pielou’s Evenness indices indicated that SL had the greatest diversity. Community structure was considered distinct at the site level as indicated by Principal Coordinates Analysis (PCoA), with more than 63.8% of the variance in community composition being explained by both synthetic axes (Supplementary Materials Figure S3).

**Figure 4:**
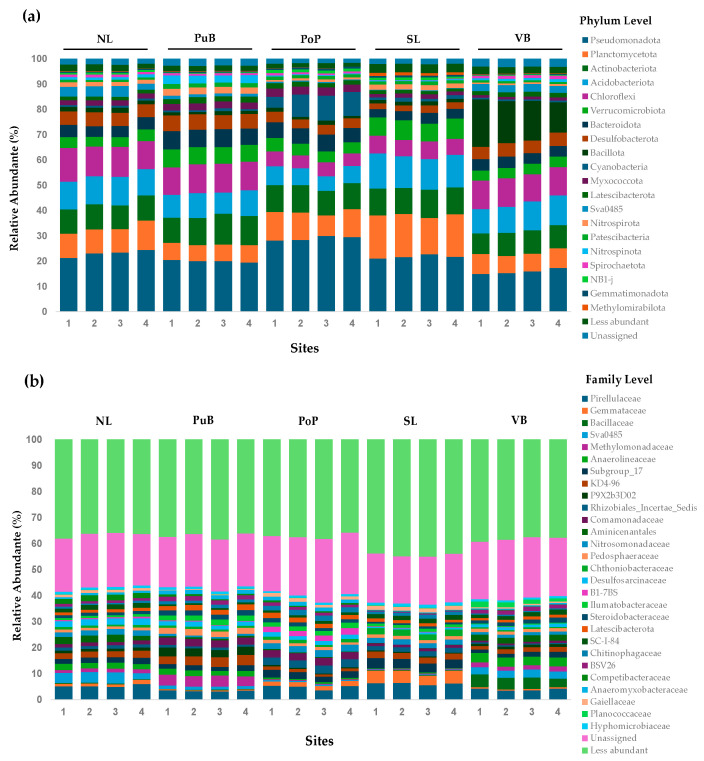
Relative abundances of bacterial communities from sediments of Lake Villarrica at (**a**) phylum and (**b**) family level. The following codes represent each replicate: North Lake (NL), Pucón City Bay (PuB), Poza Port (PoP), South Lake (SL), and Villarrica City Bay (VB). Taxonomic ranks for placeholder clades are indicated: Sva0485 (family, Anaerolineaceae), KD4-96 (order, Chloroflexota), Subgroup_17 (family, Acidobacteriota), BSV26 (family, Bacteroidota), P9 × 2b3D02 (family, Nitrospirota), B1-7BS, and SC--I--84 (family, Pseudomonadota). Classification follows the SILVA 138.1 database.

The relative abundance of the bacterial community in the sediments at the phylum (Figure 4a) and family (Figure 4b) levels shows that the top 20 most abundant phyla covered 95.19% of total relative abundance, whereas the less abundant phyla covered 2.48%; and the unassigned phylum remained at 2.32%. The most representative groups were Pseudomonadota, Planctomycetota, Actinobacteriota, Acidobacteriota, Chloroflexi, Verrucomicrobiota, Bacteroidota, Desulfobacterota, Bacillota, and Cyanobacteriota (ranging from 0.6 ± 0.1% to 29.02 ± 0.9%). Interestingly, at the less-impacted site, NL, we reported higher abundances of most of these phyla (except Bacillota) compared with VB. Conversely, VB reported higher abundances for Bacillota and Spirochaetota (1.2 ± 0.13% to 15.75 ± 2.8%) relative to other sites (0.15 ± 0.02% to 1.74 ± 0.23%).

The top 30 most abundant families accounted for 40.41% of the total relative abundance, the less abundant for 38.69%, and the unassigned families for 20.9% (Figure 4b). The most abundant families were Pirellulaceae, Acidobacteriota Subgroup_17, unclassified group *Chloroflexia* KD4-96, Comamonadaceae, Gemmataceae, uncultured delta proteobacterium Sva0485, Anaerolineaceae, Bacillaceae, Methylomonadaceae, and Nitrosomonadaceae (ranging from 0.3 ± 0.02% to 6.2 ± 0.4%). Abundances for the following families were all notably low in the VB site: Pirellulaceae, Gemmataceae, Subgroup_17, KD4-96, Rhizobiales_Incertae_Sedis, Comamonadaceae, Chthoniobacteraceae, B1-7BS, Ilumatobacteraceae, Latescibacterota, Chitinophagaceae, and Competibacteraceae (from 0.02 ± 0.01% to 4.0 ± 0.4%). In contrast, VB had high relative abundances of Planococcaceae, the Bacteroidota BSV26 family, Aminicenantales, Anaerolineaceae, and especially, Bacillaceae (from 1.7 ± 0.4% to 4.3 ± 0.7%) when compared to other sites (from 0.02 ± 0.01% to 2.5 ± 0.5%).

**Figure 5:**
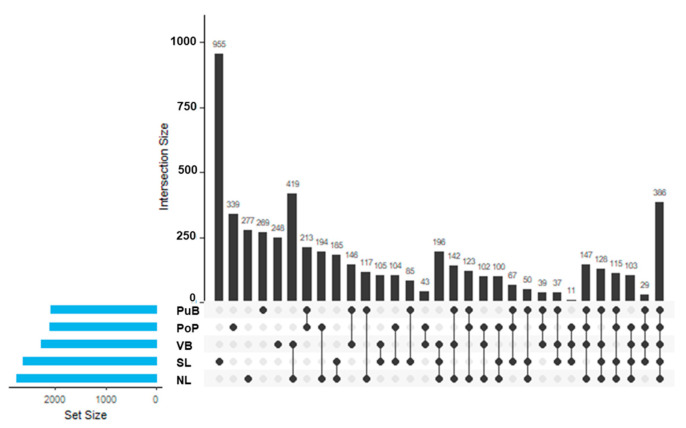
Intersection of ASVs across the sediment sites of Lake Villarrica. Horizontal bars indicate the total number of ASVs for each sampling site (NL, PuB, PoP, S, and VB) and their combinations, while vertical bars represent the number of ASVs in the category designated by the dot. Values represent the mean (*n* = 4).

Among all sites, the highest number of ASVs was found for the SL site (955 ASVs), followed by PoP (339 ASVs), NL (277 ASVs), PuB (269 ASVs), and VB (248 ASVs) (Figure 5). The UpSet analysis revealed overlap and divergence in the ASV composition among sampling sites. Similarity in ASVs was highest in the groupings VB × NL (419 ASVs) and VB × SL × NL (196 ASVs). It is found that a core microbiome of 386 ASVs was present in all five sites.

**Figure 6:**
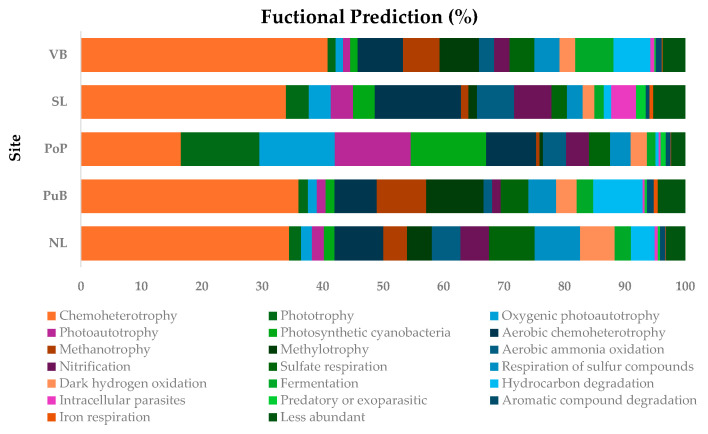
Relative abundances (%) for the top 20 major predicted metabolic and ecological functions for benthic bacterial communities in NL, PuB, PoP, SL, and VB sites analyzed with the FAPROTAX database. Values represent the mean (*n* = 4).

**Figure 7:**
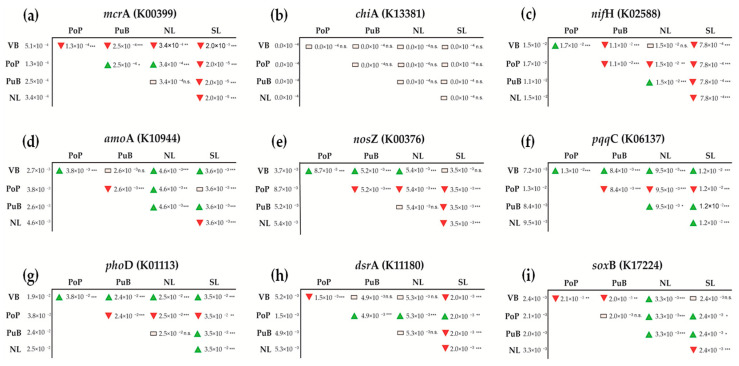
Metagenome function of specific C, N, P, and S cycling genes (**a**) *mcr*A, (**b**) *chi*A, (**c**) *nif*H, (**d**) *amo*A, (**e**) *nos*Z, (**f**) *pqq*C, (**g**) *pho*D, (**h**) *dsr*A and (**i**) *sox*B predicted by the PICRUSt2 analyses using the Kyoto Encyclopedia of Genes and Genomes (KEGG) database. Its specific metabolic annotation identifies each gene KEGG Ortholog gene (KO). Differential contrast of the gene expression and abundances among NL, PuB, PoP, S, and VB sites is represented according to their significance (* *p* < 0.05; ** *p* < 0.01; *** *p* < 0.001; n.s. no significant). The green triangle indicates an increase relative to the contrasted sample, the red triangle indicates a decrease, and the white box indicates no significant difference. Values represent the mean of replicates (*n* = 4).

Results from FAPROTAX analysis for general predicted functions are shown in Figure 6, and that of PICRUSt analysis for the prediction of the specific functional nutrient-cycling genes are shown in Figure 7. FAPROTAX results indicated that the most common bacterial functions predicted across all sites were related to chemoheterotrophy, aerobic chemoheterotrophy, respiration of sulfur compounds, and sulfate respiration (from 4.46% ± 1.9% to 32.36% ± 9.25%). The predicted performance of other functions appeared to be site-specific. For example, oxygenic photoautotrophy, photoautotrophy, photosynthetic cyanobacteria, and aerobic chemoheterotrophy (from 12.44% ± 1.9% to 13.02% ± 2.5%) were higher in PoP than the remaining sites (from 1.21% ± 0.15% to 1.31% ± 0.17%). Methanotrophy and methylotrophy were higher in PuB and VB (from 6.07% ± 0.5% to 9.48% ± 0.3%) than in NL, PoP, and SL sites (from 0.55% ± 0.15% to 4.12% ± 0.3%). Aerobic ammonia oxidation and nitrification were higher in the SL site (from 6.14% ± 0.22% to 6.17% ± 0.2%) than in NL, PoP, PuB, and VB (from 1.39% ± 0.16% to 4.7% ± 0.3%). Sulfate respiration, respiration of sulfur compounds, and dark hydrogen oxidation were higher for the NL site (from 5.79% ± 0.17% to 7.55% ± 0.4%) than for PuB, VB, and SL sites (from 1.94% ± 0.34% to 4.59% ± 0.5%). Finally, fermentation was notably higher in VB (6.32% ± 0.52%) than in the PuB, NL, SL, and PoP sites (from 1.38% ± 0.4% to 2.75% ± 0.13%).

The mean Nearest Sequenced Taxon Index (NSTI; Supplementary Materials Figure S4) for sediments found to be 0.3246 ± 0.05, which is comparable to other freshwater sediment sites (0.25–0.40) [52]. This indicates moderate phylogenetic distance from reference genomes and further suggests that PICRUSt2 predictions should be interpreted with caution. For the C-cycling genes, *mcr*A (KEGG number K00399; Figure 7a) was predicted to be higher for VB (5.1 × 10^−4^) than for PoP, PuB, SL (*p* < 0.001), and NL (*p* < 0.01) sites. *chia*A (KEGG number K13381; Figure 7b) was predicted to be very low across all sites and did not vary between sites. For the N-cycling genes, *nif*H (KEGG number K02588; Figure 7c) was predicted to be higher in PoP (1.7 × 10^−2^) than VB, PuB, SL (*p* < 0.001), and NL (*p* < 0.01). *amo*A (KEGG number K10944; Figure 7d) was predicted to be higher in NL (4.6 × 10^−3^) than in the remaining sites. Finally, the *nos*Z gene (KEGG number K00376; Figure 7e) was predicted to be significantly higher in PoP (8.7 × 10^−3^) than in all other sites (*p* < 0.001). The P-cycling genes *pqq*C (KEGG number K06137; Figure 7f) and *pho*D (KEGG number K01113; Figure 7g) were predicted to be higher in PoP (1.3 × 10^−2^ and 3.8 × 10^−2^, respectively) compared to all other sites. The S-cycling gene *dsr*A (KEGG number K11180; Figure 7h) was predicted to have similar abundances in the sites NL, VB, and PuB (from 4.9 × 10^−3^ to 5.3 × 10^−3^), which were all significantly higher (*p* < 0.001) when compared with PoP and SL (from 2.0 × 10^−3^ to 1.5 × 10^−3^). Finally, the *sox*B gene (KEGG number K17224; Figure 7i) was predicted to be higher in NL (3.3 × 10^−3^) than all other sites (*p* < 0.001).

**Figure 8:**
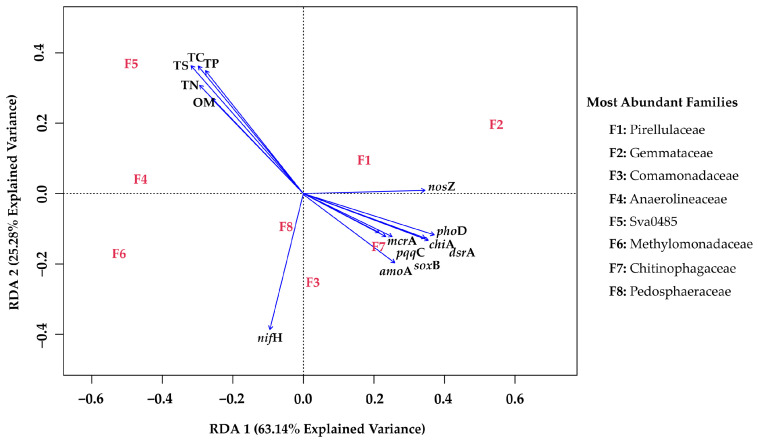
Redundancy analysis (RDA) of nutrients (TC, OM, TN, TP, and TS), absolute abundance of functional nutrient-cycling genes (*chi*A, *mcr*A, *nif*H, *amo*A, *nos*Z, *pho*D, *pqq*C, *sox*B, and *dsr*A), and the whole bacterial community of sediment samples collected from Lake Villarrica. The bacterial families most related to the variables are listed from F1 to F8.

Spearman correlations showed moderate to strong concordance between qPCR and PICRUSt2 estimates for most genes (ρ = 0.63–0.88, *p* < 0.05; Supplementary Materials Table S8), supporting the general consistency between measured and predicted functional profiles. The RDA analysis (Figure 8) demonstrated that functional nutrient-cycling genes and nutrients TS were the best predictors of data variance. The first two RDA axes together explained 88.42% of the constrained variance (Supplementary Materials Table S9). Then, in the RDA1 and RDA2 axes, explained 63.14% and 25.28% of the variability in the benthic bacterial community, respectively. A strong negative association was found between sediment nutrient content (OM, TN, TP, TS, and TC) and most nutrient-cycling genes (*amo*A, *sox*B, *pqq*C, *mcr*A, *chi*A, *dsr*A, and *pho*D). Additionally, positive associations were apparent between the bacterial families Pedosphareaceae (F8) and Comamonadaceae (F3) with the *nif*H gene; Pirellulaceae (F1) and Gammataceae (F2) with the *nos*Z gene; Sva0485 (F5) with most nutrients; and Chitinophagaceae (F7) with most genes (*amo*A, *sox*B, *pqq*C, *mcr*A, *chi*A, *dsr*A, and *pho*D).

## 4. Discussion

### 4.1. Physicochemical Status and Nutrient Contents Among Sites

Analysis of in-situ physicochemical parameters revealed significant differences between the sediment sample sites of Lake Villarrica; however, no clear trend emerged across the samples. Similar pH and temperature values were previously reported for this lake [31]. However, in the present study, lower DO values were observed, especially at the VB site (1.5 ± 0.2%), indicating high microbial activity, consistent with other findings in disturbed Chilean lakes and rivers [29,34]. The VB sediments contained considerably higher amounts of TC, TOC, OM, TN, TS, and especially TP than the other lower- to middle-nutrient-enriched sample sites (Table 1). There was an apparent clustering of sites for TS, TP, TN, TC, and TOC (and for OM, though PoP is an exception) in the series, from highest to lowest nutrients of: VB > NL > PuB | PoP | SL (Supplementary Materials Figure S1).

In general, the TC, TOC, OM, TN (expressed as % dw) and TS contents were within the lower range of values reported for some eutrophic Chinese [54,55,56,57], Greek [58], and Egyptian [59] lakes and rivers, although those studies expressed concentrations in mg kg^−1^ dw sediment (1069–4500 mg kg^−1^ C; 790–1000 mg kg^−1^ N; and 13,410 mg kg^−1^ S). Comparable results from qualitative comparison indicate that nutrient and OM enrichment in Lake Villarrica sediments is consistent with moderately eutrophic conditions. Further, TP concentrations were within the range of other well-documented eutrophic lakes (from 924 to 1597 mg kg^−1^ P) [5,60,61,62]. Collectively, these results suggest that the bed sediments are nutrient-enriched, particularly with respect to P at the VB site.

### 4.2. Relationships and Differences of Community-Level Physiological Profile (CLPP) in Sediment Sites

Biolog^®^ microwells have primarily been used to compare functional diversity rather than to characterize the community, with EcoPlates™ being most commonly used [23]. Few studies have reported on lake-bed sediments using the AWCD approach for whole microwell plates and functional categories for PM3B and PM4A. The CLPP of Lake Villarrica revealed significant variability, with low-nutrient-enriched sites being more versatile in using different nutrient sources (Figure 2 and Figure 3). It should be noted that these patterns reflect substrate utilization potentials under standardized incubation conditions rather than in-situ ecological functions and therefore indicate relative metabolic activity on plates rather than actual environmental processes. Otherwise, TTC dye reduction, which underlies color development in Biolog assays, may inhibit or underestimate the activity of specific bacterial taxa, particularly slow-growing or anaerobic taxa [38]. Additionally, incubation at 25 °C differs from in-situ lake-bottom temperatures (<20.7 °C), which may influence the relative activity profiles. Even with these limitations, this approach is a valuable tool for estimating certain ecological functions in several ecosystems. In general, evaluations of microbial communities in freshwater ecosystems using EcoPlates™ have focused on xenobiotics rather than nutrients. For example, samples from Lake Balaton in Hungary, with lower concentrations of mineral oxide nanoparticles, are more likely to utilize higher C (~2.5 AWCD) than those with higher concentrations of nanoparticles (<0.2 AWCD), revealing that their toxic effects shape the microbial community [63].

Our results are comparable to similar studies on benthic communities in the Mina Stream in Brazil, with varying degrees of heavy metal pollution [64], waste-activated sludges from dairy wastewater treatment plants [23], and constructed wastewaters [65]. Natural wetland soil samples from Lake Chaohu in China exhibited faster utilization of C sources (>0.8 AWCD) compared with soils (<0.6 AWCD) [24]. In most of these studies, bacterial communities preferentially utilized the C groups of amino acids and carboxylic acids over glucides, which is consistent with our results for Lake Villarrica. Supporting these results, benthic biofilms from Donghu, Xuanwu Lake, and Niushoushan River in China did not prefer glucose (<2.0 AWCD), but instead preferred amino acids or amines (>2.0 AWCD) [66]. The preference for these C groups over glucides may be due to a greater availability of these sources in higher nutrient sites over glucides, with a lower probability of utilizing simple glucides, as well as to differences in bacterial community structure and function [24,67].

Our results indicated that the Lake Villarrica benthic bacterial community preferred organic N sources over inorganic N sources (Figure 2b and Figure 3). While this is not normally anticipated, Kirchman [68] has pointed out that heterotrophic bacteria preferentially utilize dissolved organic N (DON), including amino acids, amines, amides, and peptides, over dissolved inorganic N (DIN) forms, such as NH_4_^+^ and NO_3_^−^, due to a lower assimilation energy cost [69]. Mena-Rivera et al. [70] showed that the rate of assimilation of N from DIN samples of the River Chew, UK, is lower than from glutamate and amino acids due to the higher energy cost of reducing NO_3_^−^ into NO_2_^−^. This is compared to the action of glutamate dehydrogenase or glutamine synthetase, which incorporate NH_4_^+^ directly into biomass. Additionally, abundant heterotrophic bacterial populations have adapted to produce proteases that depolymerize and mineralize organic nitrogen [71]. Similar adaptations have been reported for heterotrophic diazotrophs colonizing sinking particles in marine waters, which can degrade organic polymers to access N compounds [72].

The CLPP for P sources in the PM4A microwell did not exhibit a clear trend in relation to nutrient pollution levels, suggesting that the metabolism of these bacterial communities is not limited by P (Figure 2c). However, as shown in Figure 3, communities at the low-nutrient sites exhibited broader use of P compounds when compared to those at the high-nutrient sites. This was especially true for organic P compounds, including phosphonates, other P_o_ compounds, and phosphomonoesters, compared with inorganic P_i_. It has been observed that wastewater sludges preferentially utilize phosphomonoesters, likely due to the additional C made available following dephosphorylation [73]. Similarly, increased biomass yields were observed when the diazotrophs Cyanobacteriota *Prochlorococcus marinus* and *Cylindrospermopsis raciborskii* were cultured with phosphomonoesters and organic polyphosphates rather than PO_4_^3−^ [74,75]. However, diverse serovars of *Salmonella enterica* isolated from the Central California Coastal region exhibit diversity in their preference for P_o_ and P_i_ (e.g., PO_4_^3−^ and thiophosphate), demonstrating that metabolic diversity exists even within the species level [76].

In the case of CLPP for S substrate, it was evident that the low-nutrient sites were more active in utilizing S than the high-nutrient sites (Figure 2d). Although not a direct comparison, other studies have demonstrated that higher S molecule utilization occurs in nutrient-rich composts (~0.2 ACWD) when compared to nutrient-poor Himalayan desert soils (~0.5 ACWD) [77]. Unlike N and P, bacterial communities in our study prioritized the utilization of inorganic S (S_i_) groups (e.g., thiosulfates and tetrathionates) (Figure 3). This observation has been reported for isolates of *Rhizobium leguminosarum*, where S_i_ and P_i_ molecules of PM4A explained more than 33% of the variance in metabolic differentiation between bacterial strains [78]. Additionally, SRB from hypoxic sediment samples of Dangdong Bay, South Korea, were observed to change their function in response to oxygen availability [79], suggesting that REDOX coupling may be necessary for S utilization in Lake Villarrica, where Si metabolism was higher at sites with lower DO (e.g., VL).

### 4.3. Total Bacterial Community and Functional Nutrient-Cycling Gene

Total bacterial community abundances have been previously reported for polluted Chilean freshwater ecosystem (i.e., 1.8 × 10^11^ to 4.8 × 10^12^ 16S rRNA gene copy g^−1^) [5,31,34], compared to those reported here (Table 2). Most low-nutrient sites exhibited higher CLPP and lower total bacterial abundance than high-nutrient sites. Still, a significant abundance of functional nutrient-cycling genes suggests a versatile core microbiome in these sites, capable of utilizing a diverse range of nutrient molecules. In oligotrophic Lake Brienz, the abundance of the *chi*A gene was also reported to be higher than in the eutrophic Lake Zug in Switzerland, explained by an accumulation of chitin by the macroinvertebrate community under oligotrophic conditions [40]. It is important to note that bacteria belonging to the Actinobacteriota, Pseudomonadota, and Chloroflexi phyla (Figure 4a), which are more abundant in the low-nutrient sites of Lake Villarrica, are recognized as chitin degraders in freshwater ecosystems [40,80]. Our estimates for the *mcr*A gene abundance were lower than those reported for sediments of the eutrophic Everglades in Florida; the eutrophic Dianchi, Erhai, and Chahou lakes; and the Nanfei River in China (from 5 × 10^8^ to 1.7 × 10^9^ copies^−1^ and 0.7–8% of the metagenome) [21,81,82]. Elevated *mcr*A gene abundances can be attributed to OM and TOC concentrations [82,83], which are much higher in these highly nutrient-enriched sites than in our results for Lake Villarrica (Table 1).

Elevated OM content and lower oxygen availability are associated with enhanced denitrification and reduced abundance, diversity, and activity of anammox bacteria (AOB) [9,82]. This supports our finding of a lower abundance of amoA genes across all sample sites relative to *nos*Z, which in turn was lowest at the highest-nutrient site. This suggests that complete denitrification of N_2_O to N_2_ was active in the low-nutrient sites, whereas incomplete denitrification may be common in the high-nutrient sites [44]. Higher abundances of the *nos*Z gene relative to *amo*A have also been reported for polluted lakes and rivers in China and Switzerland [9,18,21,82]. However, we did not observe significant variation in the distribution of *nif*H, used as an indicator of nitrification, across the sites in Lake Villarrica. Our reported abundances for this gene were lower than those reported in other studies, for example, in North American rivers and streams (~4.5 × 10^8^) [84] and Chinese lakes [82]. Collectively, this suggests that in Lake Villarrica, the benthic bacterial community preferentially utilized DON molecules and was not limited by N.

A different case was found for *pho*D and *pqq*C genes, which are associated with P_o_ mineralization and P_i_ solubilization, respectively. Similar abundances of *pho*D have been reported in nutrient-impacted Bosten, Budi, Toltén, and Imperial Rivers, in the Araucania Region, Chile (~10^5^ to ~10^6^ gene copies) [5,34,55]. In these rivers, higher abundances of this gene at lower nutrient impacted sites indicated active mineralization of P_o,_ such as phosphomonoesters for PO_4_^3−^ supplies, which agrees with our CLPP results. However, our results did not indicate a significant difference between sites for the *pqq*C. We know of no previous reports on the *pqq*C gene as a marker for P-solubilizing bacteria (PSB) in benthic communities of lakes, although it has been well documented in terrestrial soils. There, the *pqq*C-harboring populations respond to fluctuations in TOC, TN, and pH, rather than to differences in P, which may explain our findings [85,86]. Nevertheless, the presence of the gene warrants further study in Lake Villarrica and other lakes, as well as its relationship with PSB populations [87]. Additionally, both genes were the most abundant of all functional nutrient-recycling genes, reinforcing the conclusion that the benthic microbial community plays an essential role in benthic-pelagic P cycling in Lake Villarrica.

The abundance of the *dsr*A gene, a marker for sulfate reduction by SRB, was slightly higher than that of the *sox*B gene used as a marker of sulfur oxidation by SOB across our sites. It is known that SRB is more active in anoxic and OM-rich environments. At the same time, SOB is frequently more active at oxic–anoxic interfaces (e.g., in surface lake-bed sediments), with a narrow microenvironment specialization in superficial sediment layers, which can be challenging to determine in bulk sediment samples [48]. That SOB is sensitive to oxygen availability may explain why *sox*B may be more prevalent in our low-nutrient sites, where higher DO concentrations were reported. Predominance of SRB over SOB as hypoxia increases has been observed in shallow estuarine sediments [79]. Conversely, higher abundances of *sox*B (10^5^ copies) compared to *dsr*A (10^3^ copies) were found in well-oxygenated water samples of the Pearl River [88]. Thus, *sox*B genes encoding SOX enzymes may be an important functional component of the community in Lake Villarrica, as the primary S substrate utilized was thiosulfate in the CLLP.

### 4.4. Relationship of Bacterial Community Composition and Their Predicted Functionality, and Nutrient Content and Specific Functional-Cycling-Gene Abundances

The richness and phylogenetic diversity of the bacterial communities across the five sites were not significantly different, although they exhibited unique structures (Supplementary Materials Figure S3 and Table S7). Relative abundances of the bacterial phyla Pseudomonadota, Planctomycetota, Actinobacteriota, Verrucomicrobiota, and Cyanobacteriota were higher in the less nutrient-enriched sites. This is consistent with other studies of low-nutrient lakes, including Lake Brienz [40], the Chinese Lakes Taihu, Chaohu, and Bosten [55,56,57], and the Chilean Toltén and Imperial Rivers [34]. These findings generally agree with CLPP results, as several members of these phyla are recognized as *chi*A [40], *nif*H, *nos*Z [42,82], *pho*D [5,34], and *sox*B expressing populations [88]. In contrast, the relatively nutrient-rich VB site had higher relative abundances of Bacillota, Sva0485, and Spirochaetota. Higher relative abundances of these taxa have been reported in polluted sediments from rivers, lakes, and other environments enriched in OM and sulfate, where oxygen availability is low and denitrification rates are high [21,55,82,83]. In this context, groups proposed as bioindicators of less disturbed ecosystems, including the families Pirellulaceae, Gemmataceae, Ilumatobacteraceae, Chthoniobacteaceae, KD4-96, Chitinophagaceae, Subgroup_17, B1-7BS, and the emergent Latescibacterota, which originates from the above-described phylum, were associated with our low-nutrient sites [34,55,56,57,89]. The P-cycling Bacillaceae and Planococcaceae (both from the Bacillota phylum) [82,89], the organoheterotroph Aminicenantales [90], and the organo-pollutant degrader Anaerolineaceae families [55] were all highest in our high-nutrient site VB. Finally, the higher overlap of ASVs among NL and VB (Figure 5), considering the former as the reference site for Lake Villarrica and the latter as the more polluted site, points to the special hydrodynamics of Lake Villarrica making possible the drag of superficial sediment containing specific bacterial populations [30,31], which could explain most of the similarities among previous analyses but particularly the bacterial community structure.

The most common functions predicted for Lake Villarrica community using FAPROTAX (Figure 6) were chemoheterotrophy and the respiration of sulfur compounds and sulfate. This is consistent with the observation that most OM decomposition in freshwater sediments is carried out by chemoheterotrophic bacteria [20] and aligns with the elevated CLPP observed in the EcoPlates™ with C substrates. The significant abundance of Pseudomonadota, Planctomycetota, and Desulfobacterota suggests their involvement in chemoheterotrophic processes and in the respiration of sulfur and sulfate in lake and river sediments [9,34]. In contrast, PICRUSt analysis (Figure 7) indicated a lower prediction for the *dsr*A gene in the low-nutrient PoP site, which is directly related to the respiration of sulfur and sulfate compounds. The qPCR showed higher *dsr*A gene abundances at this site. This discrepancy may be explained by the nature of PICRUSt analysis, which can perform less well in complex samples, such as sediments, and is overall less representative for environmental predictions [91]. In any case, qPCR and PICRUSt2 results were generally concordant across sites (ρ = 0.63–0.88; Supplementary Materials Table S8), supporting the inferred functional gradients while highlighting the expected uncertainty of 16S-based predictions in sediments. Conversely, the *sox*B gene was better predicted for the NL site, which matched with qPCR findings, indicating that SOB populations could actively oxidize thiosulfate (S_2_O_3_^2−^), sulfide (H_2_S), or elemental sulfur (S^0^) into sulfate (SO_4_^2−^), making it available for SRB.

Oxygenic photoautotrophy and photosynthetic cyanobacteria were notably higher in sites such as PoP, which, despite having less contaminated sediments, has frequently been a hotspot of cyanobacterial blooms in recent years [26]. Fermentation, methanotrophy, and methylotrophy were significantly higher in the OM-enriched VB site, where fermentation could introduce methanogenic bacterial populations that produce metabolic byproducts (e.g., hydrogen or acetate), thereby explaining its significant *mcr*A gene abundance [82,92]. In addition, fermentation and methylotrophy have been well associated with Aminicenatales and Desulfobacterota members, both of which are significantly abundant in the highest nutrient VB site [79,93]; PICRUSt analysis also predicted a higher abundance of this gene at this site (Figure 7a). The VB site also had a lower predicted performance of aerobic ammonia oxidation, nitrification, and denitrification, which is coincident with its lower *amo*A, *nif*H, and *nos*Z gene abundance and their PICRUSt prediction (Figure 7c,d).

Since FAPROTAX lacks any P-related function predictions, we explored PICRUSt predictions for *pho*D and *pqq*C only (Figure 7f,g). As shown by the gene abundance, *pho*D was predicted to be more prevalent in lower-nutrient sites (e.g., PoP site) where bacteria were active in phosphomonoester mineralization, presumably due to PO_4_^3−^ limitation [34]. The *pqq*C gene was also predicted to be more abundant under low-nutrient conditions, yet absolute abundances showed no significant variation across sites, indicating considerable limitations in the approaches, especially PICRUSt. In this sense, NSTI values around 0.32 (Supplementary Material Figure S4) reflect the limited genomic representation of benthic bacterial lineages in reference databases. Consequently, functional inferences from PICRUSt2 in sediments are less precise than for host-associated microbiomes. Nevertheless, comparative patterns among sites remain informative when interpreted qualitatively or alongside empirical qPCR data. Despite this, our study offers some of the first scientific insights into the environmental occurrence of this gene in lake-bed sediments.

The outcomes from RDA analysis placed nutrients and the abundance of most functional nutrient-cycling genes on opposite planes (Figure 8). This suggests that nutrient concentrations condition bacterial community total abundance (a positive association with nutrients) as well as structural and functional diversity (a negative association with nutrients) in the bed sediments. This phenomenon has been reported previously in the coastal lagoon Lake Budi, Chile, and in the North American Everglades [34,81]. Here, potential bacterial families that may serve as indicators of nutrient enrichment in the bed sediments are separated as the sensitive families (i.e., F1 to 8; Figure 8). Their abundance should be confirmed under controlled laboratory settings to inform their use as potential future indicators of sediment pollution and microbial community function.

For all bacterial communities studied in Lake Villarrica, the Chitinophagaceae family (F7) was positively correlated with functional genes, supporting the suitability as a bioindicator for less nutrient-polluted sites, consistent with its higher relative abundance in these sites. Conversely, the joint presence of Sva0485 (F5) and, to a lesser degree, the Anaerolineaceae (F4) families could be a good indicator of nutrient pollution, as they were abundant at the VB site. Pirellulaceae (F1), Gemmataceae (F2), and Pedosphareaceae (F8), which belong to the Planctomycetota phylum, are closely related to the N-cycle in less disturbed environments [9,54], as observed in our study, particularly in relation to denitrification.

## 5. Conclusions

This study provides an integrated analysis of the physiological, compositional, and functional diversity of benthic bacterial communities in Lake Villarrica in response to sediment nutrient content. Our results indicated that total bacterial abundance was highest, whereas nutrient-cycling gene abundance and diversity, as well as metabolic diversity, were lowest at the high-nutrient site (i.e., Villarrica Bay). Conversely, the site designated as a ‘reference site’ with lower anthropogenic impacts (e.g., North Lake) exhibited a lower bacterial abundance but greater community-level physiological profiles (CLPPs), functional nutrient-cycling gene abundances, and diversity, suggesting that community resilience and functional diversity were higher under lower nutrient concentration. Bacterial taxa varied with nutrient content, indicating a tendency to lose functional diversity as nutrient content is increased. This drop in functional diversity was characterized by a shift towards dominance of metabolic pathways related to methanotrophy and sulfate respiration in nutrient-rich sediments, while nitrification and the mineralization of organic phosphorus were more prevalent in low-nutrient sites. This highlights the differences as well as the importance of coupled biogeochemical processes and bacterial functions for evaluating the coupled nutrient loading, microbial responses and feedback loop.

## Figures and Tables

**Table 1:** In-situ Analysis and Nutrient Content of Sediments.

Site	pH	Temp (°C)	DO (%)	EC (µS cm^−1^)	ORP (mV)	TC (%)	TOC (%)	OM (%)	TN (%)	TP (mg kg^−1^)	TS (mg kg^−1^)
**NL**	6.7 ± 0.2 ^A^	18.4 ± 0.3 ^B^	2.0 ± 0.0 ^C^	57.4 ± 0.5 ^B^	−108.6 ± 7.7 ^AB^	3.0 ± 0.2 ^B^	2.6 ± 0.2 ^B^	4.5 ± 0.2 ^B^	0.3 ± 0.02 ^B^	677.4 ± 11.4 ^B^	568.3 ± 14.4 ^B^
**PuB**	7.3 ± 0.4 ^A^	20.7 ± 0.3 ^A^	2.5 ± 0.1 ^B^	61.3 ± 0.9 ^A^	−141.3 ± 16 ^B^	0.2 ± 0.0 ^D^	0.2 ± 0.0 ^D^	0.8 ± 0.1 ^D^	0.02 ± 0.0 ^C^	159.8 ± 4.4 ^D^	81.5 ± 2.0 ^C^
**PoP**	7.4 ± 0.2 ^A^	20.2 ± 0.7 ^A^	3.0 ± 0.1 ^A^	59 ± 0.5 ^B^	−86.9 ± 11 ^A^	0.3 ± 0.0 ^CD^	0.3 ± 0.0 ^CD^	3.7 ± 0.2 ^BC^	0.03 ± 0.0 ^C^	276.9 ± 8.0 ^C^	75.7 ± 1.4 ^C^
**SL**	6.7 ± 0.5 ^A^	18.8 ± 0.2 ^B^	2.2 ± 0.1 ^C^	58.2 ± 1.4 ^B^	−111.1 ± 29 ^AB^	0.6 ± 0.1 ^C^	0.5 ± 0.0 ^C^	1.1 ± 0.1 ^CD^	0.06 ± 0.01 ^C^	267.7 ± 9.6 ^C^	85.8 ± 1.5 ^C^
**VB**	6.7 ± 0.3 ^A^	17.5 ± 0.2 ^B^	1.5 ± 0.2 ^D^	58.1 ± 0.3 ^B^	−100.3 ± 22 ^AB^	5.4 ± 0.2 ^A^	5.3 ± 0.2 ^A^	14 ± 2.3 ^A^	0.54 ± 0.01 ^A^	1302.8 ± 12.5 ^A^	854.1 ± 15.1 ^A^

pH; Temp: temperature; DO: dissolved oxygen; EC: electrical conductivity; ORP: redox potential; TC: total carbon; TOC: total organic carbon; OM; organic matter; TN: total nitrogen; TP: total phosphorus; TS: total sulfur. Values represent the mean (*n* = 4) ± standard deviation (SD). Capital letters over SD in the same column indicate significant differences (*p* < 0.05) among sample sites per measurement, as determined by one-way ANOVA with Tukey’s honest significant difference (HSD) test. Corresponding medians and ranges (min–max) for each variable are provided in Supplementary Table S1 to account for potential non-normality in sediment chemistry distributions.

**Table 2:** Absolute Quantification of Total Bacterial Community and Functional Nutrient-Cycling Genes (*chi*A, *mcr*A, *nif*H, *amo*A, *nos*Z, *pho*D, *pqq*C, *sox*B, and *dsr*A) by qPCR.

Absolute Quantification (Gene Copy g^−1^ dw of Sediment)											
Site		16S rRNA	C		N			P		S	
			*chi*A	*mcr*A	*nif*H	*amo*A	*nos*Z	*pho*D	*pqq*C	*sox*B	*dsr*A
**NL**	Mean	7.2 × 10^10 BC^	1.2 × 10^3 A^	3.8 × 10^3 A^	2.3 × 10^4 A^	8.5 × 10^1 A^	2.4 × 10^4 A^	2.6 × 10^5 A^	1.1 × 10^5 A^	6.0 × 10^3 A^	3.0 × 10^4 C^
	SD	± 2 × 10^10^	± 2 × 10^2^	± 1 × 10^3^	± 7 × 10^3^	± 2 × 10^1^	± 4 × 10^3^	± 6 × 10^4^	± 8 × 10^4^	± 2 × 10^3^	± 3 × 10^3^
**PuB**	Mean	7.4 × 10^9 C^	4.1 × 10^2 C^	8.0 ×10^2 B^	2.6 × 10^3 B^	4.3 × 10^1 B^	6.4 × 10^2 C^	1.9 × 10^4 B^	4.2 × 10^4 A^	9.1 × 10^2 B^	8.8 × 10^3 C^
	SD	± 6 × 10^9^	± 6 × 10^1^	± 1 × 10^2^	± 1 × 10^3^	± 1 × 10^1^	± 9 × 10^0^	± 8 × 10^3^	± 1 × 10^4^	± 8 × 10^1^	± 1 × 10^3^
**PoP**	Mean	1.5 × 10^11 B^	7.8 × 10^2 B^	3.2 × 10^3 B^	1.5 × 10^4 AB^	4.0 × 10^1 B^	6.3 × 10^3 B^	3.0 × 10^5 A^	1.2 × 10^5 A^	8.1 × 10^3 A^	2.3 × 10^4 A^
	SD	± 8 × 10^10^	± 5 × 10^0^	± 1 × 10^2^	± 3 × 10^3^	± 9 × 10^0^	± 8 × 10^1^	± 1 × 10^5^	± 1 × 10^4^	± 1 × 10^3^	± 3 × 10^3^
**SL**	Mean	8.1 × 10^10 BC^	6.0 × 10^2 BC^	5.8 × 10^2 C^	1.5 × 10^4 AB^	6.7 × 10^1 B^	5.1 × 10^3 BC^	2.5 × 10^5 A^	3.9 × 10^4 A^	2.5 × 10^3 B^	1.5 × 10^4 B^
	SD	± 3 × 10^9^	± 8 × 10^1^	± 5 × 10^1^	± 7 × 10^3^	± 2 × 10^1^	± 1 × 10^2^	± 4 × 10^4^	± 1 × 10^4^	± 4 × 10^2^	± 3 × 10^3^
**VB**	Mean	2.9 × 10^11 A^	9.1 × 10^1 D^	2.1 × 10^3 AB^	2.7 × 10^3 B^	1.4 × 10^1 B^	1.3 × 10^3 BC^	3.6 × 10^3 B^	2.4 × 10^4 A^	1.5 × 10^2 B^	4.7 × 10^3 C^
	SD	± 6 × 10^10^	± 2 × 10^1^	± 8 × 10^2^	± 2 × 10^2^	± 2 × 10^0^	± 7 × 10^1^	± 2 × 10^3^	± 1 × 10^4^	± 6 × 10^1^	± 3 × 10^2^

Quantification by qPCR with an R^2^ from 98.2 to 99.9 and an efficiency 91.2% to 108.7%. Values represent the mean (*n* = 4) ± standard deviation (SD). Capital letters over SD in the same column indicate significant differences (*p* < 0.05) among sample sites per measurement, as determined by one-way ANOVA with Tukey’s honest significant difference (HSD) test.

## Data Availability

The original data presented in the study are openly available in the Sequence Read Archive (SRA) of the National Center for Biotechnology Information (NCBI) at https://www.ncbi.nlm.nih.gov/sra (accessed on 11 April 2025) under accession number PRJNA864849.

## Supplementary Materials

The following supporting information can be downloaded at https://www.mdpi.com/article/10.3390/microorganisms13112544/s1, Table S1. Medians and ranges (minimum–maximum) for all nutrient parameters; Table S2: Media recipes used for the CLPP approach; Table S3: Kinetic Curve AWCD from the whole plate at each time point; Table S4: Area under the curve (AUC) of Average Well Color Development (AWCD); Table S5: Individual AWCD at endpoint AWCD (96 h) per C, N, P, and S source and samples.; Table S6: Primer sets and PCR conditions used for the quantification of bacterial functional genes; Table S7: Richness and Alpha diversity of sediment samples from Lake Villarrica; Table S8: Spearman’s rank correlation analysis between qPCR-measured and PICRUSt2-predicted gene abundances across sampling sites; Table S9: Results of Inertia, proportion constrained, r^2^, adjusted r^2^, and P-value of each variable included in the partial redundancy analysis (RDA) of this study; Figure S1: Standardized (z-score) plots illustrating the nutrient gradient among sample sites; Figure S2: Substrate layout and functional category mapping; Figure S3: Beta diversity; Figure S4. NSTI (Nearest Sequenced Taxon Index). Reference [94] is cited in the Supplementary Materials.
