# Supplementary material for: Integrated Assessment of Benthic Bacterial Community Physiology, Structure, and Function Across C, N, P, and S Gradients in Lake Villarrica Sediments, Chile
Source: Microorganisms. 2025 Nov 7;13(11):2544. doi: 10.3390/microorganisms13112544 (PMC12654752; doi:10.3390/microorganisms13112544)
Supplement: Supplementary file 1 [file microorganisms-13-02544-s001.zip › Supplementary_Material_MCB1.pdf]

Supplementary material

Tables and Figures

Integrated Assessment of Benthic Bacterial Community Physiology, Structure, and Function Across C, N, P, and S Gradients in Lake Villarrica Sediments, Chile

Tay Ruiz-Gil [1], Sebastián Elgueta [2], Giovanni Larama [3], Joaquín-Ignacio Rilling [4], Anthony Hollenback [5], Deb P. Jaisi [5], Diego Valdebenito [1], Bryan M. Spears [6], and Marco A. Campos [1] *

1. Laboratorio de Investigación Interdisciplinaria en Microbiología Aplicada (LIMA), Centro de Investigación en Ciencias de la Salud, Departamento de Procesos Diagnósticos y Evaluación, Facultad de Ciencias de la Salud, Universidad Católica de Temuco, Temuco 4810399, Chile.
2. Escuela de Nutrición y Dietética, Facultad de Ciencias de la Rehabilitación y Calidad de Vida, Universidad San Sebastián, Sede Los Leones, Providencia, Chile.
3. Biocontrol Research Laboratory, Universidad de La Frontera, Temuco, 4780000, Chile.
4. Millennium Institute Center for Genome Regulation (MI-CGR), Santiago, Chile
5. Department of Plant and Soil Sciences, University of Delaware, Newark, DE 19716, USA
6. UK Centre for Ecology & Hydrology, Bush Estate, Penicuik, Midlothian, EH26 0QB, United Kingdom.

* Correspondence: marco.campos@uct.cl

Table S1. Medians and ranges (minimum–maximum) for all nutrient parameters (TC, TOC, OM, TN, TP, TS).

Nutrient	Site	Mean ± SD	Median	Range (Min–Max)
TC (%)	NL	2.99 ± 0.23	2.99	2.75 – 3.21
	PuB	0.21 ± 0.01	0.21	0.20 – 0.22
	PoP	0.30 ± 0.03	0.3	0.27 – 0.32
	SL	0.57 ± 0.05	0.59	0.51 – 0.61
	VB	5.36 ± 0.18	5.32	5.19 – 5.55
TOC (%)	NL	2.56 ± 0.19	2.56	2.37 – 2.75

	PuB	0.20 ± 0.03	0.19	0.17 – 0.23
	PoP	0.30 ± 0.03	0.3	0.27 – 0.33
	SL	0.50 ± 0.03	0.5	0.47 – 0.53
	VB	5.28 ± 0.24	5.28	5.05 – 5.55
**OM (%)**	NL	4.54 ± 0.20	4.52	4.32 – 4.76
	PuB	0.81 ± 0.10	0.82	0.70 – 0.91
	PoP	3.67 ± 0.19	3.68	3.46 – 3.90
	SL	1.14 ± 0.07	1.13	1.08 – 1.23
	VB	13.98 ± 2.28	14	11.79 – 16.36
**TN (%)**	NL	0.307 ± 0.031	0.3	0.28–0.34
	PuB	0.020 ± 0.010	0.02	0.01–0.03
	PoP	0.027 ± 0.006	0.03	0.02–0.03
	SL	0.057 ± 0.015	0.06	0.04–0.07
	VB	0.540 ± 0.020	0.54	0.52–0.56
**TP (mg kg^{-1})**	NL	677.4 ± 11.4	675.6	667.0 – 689.6
	PuB	159.8 ± 4.4	158.3	156.3 – 164.7
	PoP	276.9 ± 8.0	277.7	268.5 – 284.4
	SL	267.7 ± 9.6	264.9	259.9 – 278.4
	VB	1302.8 ± 12.5	1304.3	1289.6 – 1314.5
**TS (mg kg^{-1})**	NL	568.3 ± 14.4	568.5	553.7 – 582.6
	PuB	81.5 ± 2.0	81.3	79.7 – 83.6
	PoP	75.7 ± 1.4	75.2	74.7 – 77.2
	SL	85.8 ± 1.5	86.4	84.1 – 86.9
	VB	854.1 ± 15.1	859.5	837.0 – 865.6

Table S2. Media recipes used for the CLPP approach.

M9 minimal medium is a classical bacterial growth broth (Miller, et al., 1972) characterized by its low autofluorescence.

Reference:

Miller, J.H. (1972). Experiments in Molecular Genetics. Cold Spring Harbor Laboratory, Cold Spring Harbor, New York.

ATCC Medium: MD-6032 M9 Medium recipe

M9 salts

1. Sodium phosphate dibasic (anhydrous), Na_2HPO_4………..6.0 g

2. Potassium phosphate monobasic, KH_2PO_4…..……………...3.0 g

3. Sodium chloride, NaCl……………………………..…………..0.5 g

4. Ammonium chloride, NH_4Cl……………………….………1.0 g

DI Water, dH_2O to 1 liter

Adjust pH to 7.4

Dissolve M9 salts and autoclave at 121ºC.

Add the following filter sterilized solutions:

5. 1 M Calcium chloride, $CaCl_2$ Solution ……………………..10 ml

6. 1 M Magnesium sulfate, $MgSO_4$ Solution……………………1 ml

7. Succinate(20Mm)-Citrate(2uM) (20% Solution)*……………..1 ml

Dispense complete medium into the appropriate vessel.

*Using succinate/citrate instead of glucose for M9 broth incubation allows study-specific metabolic processes that occur in response to a switch to these less-preferred energy substrates, since glucose represses the activity of several enzymes

Modifications for C, N, P, and S deprived media

	CM9	NM9	PM9	SM9
Na_2HPO_4	x	x		x
KH_2PO_4	x	x		x
NaCl	x	x	x	x
NH_4Cl	x		x	x
$CaCl_2$	x	x	x	x
$MgSO_4$	x	x	x	
Succinate-Citrate		x	x	x

Table S3. Kinetic Curve AWCD from the whole plate at each time point

ST3a. C sources (Biolog® EcoPlates™)

Time (h)	NL				PuB				PoP				SL				VB			
**0**	0.00	±	0.00	A	0	±	0	A	0	±	0	A	0	±	0	A	0	±	0	A
**3**	0.01	±	0.00	A	0.04	±	0.01	A	0.01	±	0.00	A	0.02	±	0.03	A	0.01	±	0.01	A
**6**	0.01	±	0.00	D	0.18	±	0.01	A	0.01	±	0.00	D	0.09	±	0.02	C	0.14	±	0.02	B
**10**	0.10	±	0.01	E	0.66	±	0.02	A	0.17	±	0.03	D	0.28	±	0.02	C	0.61	±	0.01	B
**24**	0.21	±	0.01	D	0.83	±	0.01	A	0.41	±	0.02	C	0.44	±	0.00	C	0.73	±	0.01	B
**30**	0.34	±	0.01	E	0.92	±	0.01	A	0.58	±	0.01	D	0.68	±	0.02	C	0.80	±	0.01	B
**48**	0.81	±	0.02	C	0.97	±	0.02	A	0.92	±	0.01	B	0.78	±	0.01	C	0.89	±	0.01	B
**54**	0.95	±	0.03	B	0.99	±	0.02	B	1.06	±	0.02	A	0.86	±	0.01	C	0.97	±	0.01	B
**72**	1.09	±	0.03	B	1.02	±	0.01	C	1.20	±	0.03	A	0.93	±	0.01	D	1.02	±	0.02	C
**78**	1.14	±	0.02	B	1.03	±	0.01	C	1.23	±	0.02	A	0.94	±	0.01	D	1.02	±	0.02	C
**96**	1.15	±	0.01	B	1.03	±	0.02	C	1.23	±	0.03	A	0.94	±	0.01	D	1.03	±	0.02	C

ST3b. N sources (Biolog® PM3B)

Time (h)	NL				PuB				PoP				SL				VB			
**0**	0.00	±	0.00	A	0.00	±	0.00	A	0.00	±	0.00	A	0.00	±	0.00	A	0.00	±	0.00	A
**12**	0.02	±	0.01	A	0.03	±	0.01	A	0.04	±	0.01	A	0.03	±	0.01	A	0.03	±	0.00	A
**24**	0.10	±	0.01	AB	0.14	±	0.00	A	0.08	±	0.02	B	0.08	±	0.01	B	0.13	±	0.01	A
**42**	0.30	±	0.01	B	0.38	±	0.01	A	0.21	±	0.01	D	0.26	±	0.01	C	0.31	±	0.01	B
**48**	0.40	±	0.01	B	0.47	±	0.00	A	0.28	±	0.01	D	0.32	±	0.01	C	0.39	±	0.01	B
**60**	0.47	±	0.01	B	0.53	±	0.01	A	0.36	±	0.01	D	0.41	±	0.01	C	0.46	±	0.02	B
**66**	0.51	±	0.01	B	0.56	±	0.01	A	0.40	±	0.01	E	0.44	±	0.01	D	0.49	±	0.01	C

**72**	0.53 ± 0.01 B	0.58 ± 0.01 A	0.44 ± 0.01 D	0.44 ± 0.02 D	0.51 ± 0.02 C
**84**	0.55 ± 0.01 B	0.60 ± 0.02 A	0.46 ± 0.01 C	0.44 ± 0.02 C	0.52 ± 0.02 B
**90**	0.55 ± 0.01 B	0.60 ± 0.02 A	0.46 ± 0.01 C	0.45 ± 0.03 C	0.52 ± 0.03 B
**96**	0.55 ± 0.01 B	0.60 ± 0.02 A	0.46 ± 0.01 C	0.45 ± 0.02 C	0.52 ± 0.02 B

ST3c. P sources (Biolog® PM4A)

Time (h)	NL	PuB	PoP	SL	VB
**0**	0.00 ± 0.00 A	0.00 ± 0.00 A	0.00 ± 0.00 A	0.00 ± 0.00 A	0.00 ± 0.00 A
**12**	0.04 ± 0.00 A	0.02 ± 0.00 B	0.01 ± 0.01 B	0.01 ± 0.01 B	0.03 ± 0.01 AB
**24**	0.37 ± 0.01 A	0.28 ± 0.01 B	0.12 ± 0.00 E	0.20 ± 0.00 D	0.25 ± 0.01 C
**42**	0.66 ± 0.01 A	0.61 ± 0.01 B	0.37 ± 0.01 E	0.49 ± 0.01 D	0.55 ± 0.01 C
**48**	0.75 ± 0.01 A	0.67 ± 0.01 B	0.50 ± 0.01 E	0.56 ± 0.01 D	0.62 ± 0.01 C
**60**	0.79 ± 0.01 A	0.71 ± 0.02 B	0.60 ± 0.01 D	0.58 ± 0.01 D	0.66 ± 0.01 C
**66**	0.81 ± 0.01 A	0.73 ± 0.02 B	0.64 ± 0.02 CD	0.61 ± 0.01 D	0.69 ± 0.01 C
**72**	0.82 ± 0.02 A	0.75 ± 0.01 B	0.67 ± 0.01 C	0.65 ± 0.02 C	0.69 ± 0.02 C
**84**	0.82 ± 0.02 A	0.75 ± 0.01 B	0.67 ± 0.03 D	0.65 ± 0.01 D	0.70 ± 0.02 C
**90**	0.81 ± 0.01 A	0.75 ± 0.01 B	0.67 ± 0.02 D	0.65 ± 0.01 D	0.70 ± 0.03 C
**96**	0.81 ± 0.03 A	0.76 ± 0.01 B	0.67 ± 0.01 D	0.65 ± 0.01 D	0.70 ± 0.03 C

ST3d. Sulfurated Microwell Plate (Biolog® PM4A)

Time (h)	NL	PuB	PoP	SL	VB
**0**	0.00 ± 0.00 A	0.00 ± 0.00 A	0.00 ± 0.00 A	0.00 ± 0.00 A	0.00 ± 0.00 A
**12**	0.02 ± 0.00 A	0.02 ± 0.02 A	0.02 ± 0.00 A	0.01 ± 0.00 A	0.02 ± 0.00 A
**24**	0.24 ± 0.00 A	0.25 ± 0.02 A	0.08 ± 0.01 C	0.15 ± 0.01 B	0.16 ± 0.00 B
**42**	0.61 ± 0.01 A	0.64 ± 0.01 A	0.38 ± 0.01 D	0.46 ± 0.03 C	0.52 ± 0.02 B
**48**	0.66 ± 0.00 A	0.67 ± 0.01 A	0.51 ± 0.01 C	0.57 ± 0.03 B	0.57 ± 0.01 B
**60**	0.71 ± 0.01 A	0.69 ± 0.01 A	0.61 ± 0.01 B	0.61 ± 0.04 B	0.60 ± 0.02 B
**66**	0.72 ± 0.01 A	0.72 ± 0.01 A	0.63 ± 0.01 B	0.64 ± 0.01 B	0.62 ± 0.02 B
**72**	0.74 ± 0.01 A	0.73 ± 0.01 A	0.64 ± 0.01 B	0.64 ± 0.03 B	0.63 ± 0.03 B
**84**	0.73 ± 0.02 A	0.73 ± 0.01 A	0.64 ± 0.03 B	0.64 ± 0.04 B	0.63 ± 0.01 B
**90**	0.73 ± 0.01 A	0.74 ± 0.03 A	0.64 ± 0.01 B	0.65 ± 0.02 B	0.63 ± 0.02 B
**96**	0.74 ± 0.03 A	0.74 ± 0.02 A	0.64 ± 0.03 B	0.65 ± 0.02 B	0.63 ± 0.02 C

Table S4. Area under the curve (AUC) of Average Well Color Development (AWCD) for C sources (Biolog® EcoPlates™), N sources (Biolog® PM3B), P sources (Biolog® PM4A), and S sources (Biolog® PM4A) at endpoint AWCD (96 hrs).

	AUC (OD·h) ± SD			
**Site**	**EcoPlates™ (C sources)**	**PM3B (N sources)**	**PM4A (P sources)**	**PM4A (S sources)**
**NL**	65.3 ± 1.2	43.4 ± 1.1	70.8 ± 1.2	63.2 ± 1.1
**PuB**	83.4 ± 1.4	48.5 ± 1.3	64.4 ± 1.3	63.4 ± 1.2
**PoP**	76.8 ± 1.3	34.6 ± 1.0	53.9 ± 1.1	52.5 ± 1.0
**SL**	66.0 ± 1.1	36.2 ± 1.1	54.4 ± 1.0	54.5 ± 1.1
**VB**	78.8 ± 1.3	42.0 ± 1.2	59.5 ± 1.2	54.0 ± 1.1

Values represent the mean (n = 3) ± standard deviation (SD). Corresponding AUC values per site (mean ± 95% CI) are provided. Higher AUC indicates greater overall metabolic activity.

Table S5. Individual AWCD at endpoint AWCD (96 hrs) per C, N, P, and S source and samples.

**ST4a. Carbonated Microwell Plate (Biolog® EcoPlates™)**						
**Functional classification**	**Individual Source**	**Absorbance***				
		**NL**	**PuB**	**PoP**	**SL**	**VB**
**Amino acids**	L-Arginine	2.43	2.16	2.35	2.14	1.94
	L-Asparagine	2.48	1.99	2.28	1.91	1.95
	L-Phenylalanine	2.41	2.16	2.39	2.23	2.06
	L-Serine	2.17	2.17	2.37	1.88	1.97
	β-Hydroxy-Glycyl-L-Glutamic Acid	0.32	0.76	0.42	0.56	0.27
	L-Threonine	1.93	2.04	2.29	2.11	1.88
**Amines**	Phenylethylamine	1.21	2.08	1.71	1.30	1.84
	Putrescine	1.23	2.03	1.66	1.21	1.69
**Glucides**	D-Mannitol	2.56	0.68	2.34	0.43	0.62
	Glucose-1-Phosphate	0.20	0.00	0.28	0.04	0.03
	D,L-α-Glycerol Phosphate	0.27	0.06	0.36	0.03	0.03
	β-Methyl-D-Glucoside	0.15	0.03	0.28	0.01	0.04

	D-Galactonic Acid γ-Lactone	1.95	1.93	2.22	1.22	1.83
	i-Erythritol	0.16	0.02	0.29	0.07	0.04
	D-Xylose	2.06	0.57	2.21	1.11	0.50
	N-Acetyl-D-Glucosamine	2.44	1.99	2.39	1.21	2.05
	D-Cellobiose	0.15	0.24	0.25	0.29	0.17
	α-D-Lactose	0.17	0.07	0.26	1.11	0.04
**Carboxylic acids**	D-Glucosaminic Acid	2.23	1.92	2.07	2.20	1.79
	D-Malic Acid	0.36	0.21	1.92	0.47	0.15
	Itaconic Acid	0.16	0.06	0.44	0.05	0.02
	Pyruvic Acid Methyl Ester	2.03	0.10	2.08	0.08	0.07
	D-Galacturonic Acid	2.44	1.90	2.32	1.95	1.65
	α-Keto Butyric Acid	0.13	0.01	0.22	0.05	0.04
	γ-Amino Butyric Acid	2.30	2.08	2.03	2.17	1.90
**Phenolic compounds**	2-Hydroxy Benzoic Acid	0.15	0.03	0.25	0.07	0.04
	4-Hydroxy Benzoic Acid	2.08	1.22	2.16	1.19	1.65
**Polymers**	Tween 80	1.91	1.33	2.00	0.91	1.28
	Tween 40	1.95	1.45	2.11	1.02	1.38
	α-Cyclodextrin	0.18	0.15	0.29	0.16	0.06
	Glycogen	0.25	0.07	0.29	0.05	0.03

ST4b. Nitrogenated Microwell Plate (Biolog® PM3B)

Functional classification	Individual Source	Absorbance*				
		NL	PuB	PoP	SL	VB
**Ammonia**		0.48	0.74	0.46	0.42	0.44
**Nitrite**		0.40	0.45	0.44	0.47	0.55
**Nitrate**		0.64	0.84	0.48	0.48	0.66
**Amino Acids and Derivatives**	L-Alanine	0.65	0.72	0.47	0.55	0.68
	L-Arginine	0.74	1.27	0.74	0.63	1.03
	L-Asparagine	0.76	0.66	0.63	0.59	0.84
	L-Aspartic acid	0.58	0.65	0.58	0.57	0.65
	L-Cysteine	0.68	0.04	0.58	0.16	0.69
	L-Glutamic acid	0.54	0.48	0.36	0.37	0.50
	L-Glutamine	0.73	0.93	0.67	0.64	0.87
	Glycine	0.48	0.52	0.46	0.52	0.64
	L-Histidine	0.84	0.93	0.65	0.65	0.99
	L-Isoleucine	0.84	0.63	0.47	0.57	0.83
	L-Leucine	0.63	0.57	0.56	0.42	0.60
	L-Lysine	0.84	0.99	0.78	0.67	0.97
	L-Methionine	0.48	0.75	0.41	0.61	0.88
	L-Phenylalanine	0.42	0.84	0.42	0.50	0.62
	L-Proline	0.60	0.43	0.41	0.42	0.62

	L-Serine	0.58	0.63	0.46	0.48	0.65
	L-Threonine	0.63	0.62	0.38	0.42	0.73
	L-Tryptophan	0.55	0.27	0.46	0.39	0.86
	L-Tyrosine	0.29	0.18	0.16	0.35	0.31
	L-Valine	0.63	0.55	0.61	0.35	0.65
	D-Alanine	0.64	0.55	0.46	0.49	0.56
	D-Asparagine	0.84	0.85	0.77	0.61	0.82
	D-Aspartic acid	0.73	0.46	0.64	0.44	0.70
	D-Glutamic acid	0.73	0.66	0.68	0.50	0.68
	D-Lysine	0.44	1.07	0.02	0.52	0.73
	D-Serine	0.23	0.48	0.54	0.27	0.65
	D-Valine	0.59	0.63	0.48	0.54	0.64
	L-Citrulline	0.74	0.78	0.85	0.59	1.06
	L-Homoserine	0.32	0.56	0.05	0.34	0.05
	L-Ornithine	0.75	0.88	0.69	0.65	0.94
	N-Acetyl-L-Glutamic acid	0.39	0.23	0.34	0.45	0.49
	N-Phthaloyl-L-Glutamic acid	0.08	0.04	0.01	0.05	0.04
	L-Pyroglutamic acid	0.76	0.69	0.66	0.55	0.52
	DL-a-Amino-N-Butyric acid	0.41	0.35	0.47	0.44	0.44
	g-Amino-N-Butyric acid	0.58	0.44	0.47	0.41	0.63
	e-Amino-N-Caproic acid	0.02	0.05	0.01	0.04	0.06
	DL-a-Amino-Caprylic acid	0.39	0.05	0.30	0.05	0.04
	d-Amino-N-Valeric acid	0.77	0.36	0.37	0.54	0.68
	a-Amino-N-Valeric acid	0.68	0.74	0.51	0.48	0.68
	D-Glucosamine	0.49	0.37	0.30	0.04	0.01
	D-Galactosamine	0.04	0.08	0.02	0.04	0.02
	D-Mannosamine	0.08	0.06	0.03	0.04	0.01
	N-Acetyl-D-Glucosamine	0.64	0.60	0.61	0.54	0.01
	N-Acetyl-D-Galactosamine	0.04	0.04	0.04	0.07	0.15
	N-Acetyl-D-Mannosamine	0.05	0.04	0.02	0.05	0.05
**Amines and derivatives**	Hydroxylamine	0.05	0.01	0.01	0.02	0.02
	Methylamine	0.35	0.71	0.01	0.03	0.74
	N-Amylamine	0.50	0.49	0.35	0.35	0.53
	N-Butylamine	0.35	0.34	0.02	0.56	0.50
	Ethylamine	0.22	0.47	0.03	0.03	0.26
	Ethanolamine	0.64	0.74	0.70	0.68	0.78
	Ethylenediamine	0.04	0.03	0.01	0.03	0.03
	Putrescine	0.82	0.35	0.84	0.65	0.73
	Agmatine	0.74	0.57	0.43	0.79	0.84
	Histamine	0.85	0.79	0.66	0.04	0.01
	b-Phenylethylamine	0.44	0.31	0.40	0.35	0.01
	Tyramine	0.67	0.58	0.56	0.50	0.01
**Amides and derivatives**	Acetamide	0.44	0.51	0.37	0.38	0.01

	Formamide	0.17	0.20	0.05	0.06	0.01
	Glucuronamide	0.50	0.35	0.24	0.28	0.01
	DL-Lactamide	0.69	0.16	0.57	0.54	0.02
	Urea	0.86	0.98	0.66	0.67	0.94
	Biuret	0.87	0.94	0.65	0.74	0.95
**Nucleobases and derivatives**	Adenine	0.76	0.59	0.58	0.70	0.64
	Adenosine	0.75	0.80	0.70	0.56	0.71
	Cytidine	0.69	0.17	0.44	0.60	0.70
	Cytosine	0.84	0.94	0.73	0.64	0.85
	Guanine	0.43	0.72	0.52	0.58	0.70
	Guanosine	0.67	0.05	0.64	0.40	0.71
	Thymine	0.49	0.73	0.50	0.55	0.75
	Thymidine	0.06	0.04	0.02	0.04	0.04
	Uracil	0.72	0.88	0.55	0.57	0.73
	Uridine	0.64	0.34	0.34	0.32	0.73
	Inosine	0.65	0.98	0.82	0.41	0.65
	Xanthine	0.33	0.04	0.08	0.05	0.04
	Xanthosine	0.81	0.73	0.89	0.59	0.74
	Uric acid	0.79	0.91	0.65	0.69	0.87
	Alloxan	0.20	0.06	0.02	0.07	0.07
	Allantoin	0.73	0.75	0.69	0.65	0.76
	Parabanic acid	0.22	0.07	0.13	0.43	0.09
**Peptides**	Ala-Asp	0.80	0.40	0.56	0.63	0.66
	Ala-Gln	0.81	0.81	0.79	0.58	0.66
	Ala-Glu	0.83	0.84	0.66	0.63	0.76
	Ala-Gly	0.77	0.96	0.65	0.67	0.74
	Ala-His	0.74	0.84	0.61	0.62	0.83
	Ala-Leu	0.74	0.72	0.59	0.65	0.75
	Ala-Thr	0.94	0.91	0.70	0.44	0.63
	Gly-Asn	0.74	0.64	0.54	0.59	0.77
	Gly-Gln	0.64	0.64	0.54	0.63	0.70
	Gly-Glu	0.57	0.77	0.61	0.57	0.60
	Gly-Met	0.75	0.93	0.58	0.46	0.78
	Met-Ala	0.75	0.60	0.50	0.41	0.68

ST4c. Phosphorated Microwell Plate (Biolog® PM4A)

Functional classification	Individual Source	Absorbance*				
		NL	PuB	PoP	SL	VB
**Phosphate**		0.74	0.77	0.66	0.68	0.68
**Polyphosphates**	Pyrophosphate	0.78	0.86	0.73	0.69	0.70
	Trimetaphosphate	0.76	0.80	0.76	0.75	0.72
	Tripolyphosphate	0.99	0.88	0.70	0.73	0.72

**Hypophosphates and Thiphosphates**	Hypophosphite	0.73	0.67	0.71	0.44	0.41
	Thiophosphate	0.88	0.85	0.73	0.73	0.73
	Dithiophosphate	0.76	0.84	0.81	0.76	0.71
**Phosphomonoesters**	D, L - α -Glycerol Phosphate	0.88	1.24	0.73	0.76	0.72
	β-Glycerol Phosphate	0.94	0.85	0.58	0.84	0.87
	Carbamyl Phosphate	0.92	0.91	0.76	0.85	0.82
	D - 2 - Phospho-Glyceric Acid	0.95	0.85	0.78	0.76	0.81
	D - 3 - Phospho -Glyceric Acid	0.78	0.92	0.83	0.74	0.85
	Phosphoenol Pyruvate	0.76	0.67	0.73	0.78	0.75
	Phospho-Glycolic Acid	0.95	0.95	0.81	0.79	0.83
	D - Glucose - 1 -Phosphate	0.77	0.72	0.58	0.78	0.93
	D - Glucose - 6 -Phosphate	1.07	1.09	0.74	0.66	0.80
	2 - Deoxy - D -Glucose 6 - Phosphate	0.93	0.73	0.46	0.63	0.86
	D -Glucosamine - 6 Phosphate	1.21	0.76	0.65	0.76	0.81
	6 - Phospho -Phospho-Gluconic Acid	0.93	0.95	0.76	0.72	0.93
	D - Mannose - 1 Phosphate	0.79	0.87	0.77	0.69	0.64
	D - Mannose - 6 Phosphate	0.90	0.78	0.84	0.74	0.84
	O - Phospho - D -Serine	1.02	1.08	0.67	0.70	0.80
	O - Phospho - L -Serine	1.00	1.36	0.87	0.79	0.84
	O - Phospho - L- Threonine	0.92	1.04	0.76	0.73	0.79
	O - Phospho - D- Tyrosine	0.85	0.88	0.89	0.77	0.74
	O - Phospho - L- Tyrosine	0.86	0.82	0.81	0.75	0.95
	Adenosine - 2′ -monophosphate	0.84	0.72	0.90	0.74	0.72
	Adenosine-3′-monophosphate	0.75	0.53	0.97	0.64	0.49
	Adenosine - 5′ -monophosphate	0.92	0.60	1.00	0.67	0.81
	Adenosine -2′, 3′ - cyclic monophosphate	0.68	0.76	0.48	0.73	0.48
	Adenosine-3′, 5′-cyclic monophosphate	0.73	0.64	0.55	0.67	0.44
	Guanosine - 2′ -monophosphate	0.99	0.63	0.54	0.72	0.86
	Guanosine - 3′ -monophosphate	0.96	0.88	0.72	0.76	0.84
	Guanosine - 5′ -monophosphate	0.86	0.84	0.72	0.73	0.97
	Guanosine -2′, 3′ - cyclic monophosphate	1.00	0.78	0.74	0.46	0.83
	Guanosine-3 -3′, 5′ - cyclic monophosphate	0.87	0.61	0.64	0.45	0.50
	Cytidine - 2′ -monophosphate	1.05	0.93	0.84	0.76	0.93
	Cytidine - 3′ -monophosphate	0.95	0.82	0.44	0.48	0.62
	Cytidine - 5′ -monophosphate	0.86	0.84	0.71	0.75	0.84
	Cytidine - 2′,3′ cyclic monophosphate	0.86	0.73	0.68	0.75	0.73

	Cytidine - 3′ ,5′ - cyclic monophosphate	0.86	0.89	0.65	0.64	0.52
	Uridine - 2′ -monophosphate	1.05	1.11	0.85	0.65	0.87
	Uridine - 3′ -monophosphate	0.92	0.87	0.81	0.54	0.73
	Uridine - 5′ -monophosphate	0.97	0.72	0.72	0.76	0.85
	Uridine - 2′,3′ -cyclic monophosphate	1.00	0.73	0.86	0.79	0.88
	Uridine - 3′,5′ -cyclic monophosphate	0.89	0.63	0.63	0.84	0.75
	Thymidine - 3 ′ - monophosphate	0.95	0.97	0.66	0.75	0.82
	Thymidine - 5 ′ - monophosphate	0.93	0.96	0.64	0.67	0.92
	Thymidine 3′, 5′ - cyclic monophosphate	0.60	0.63	0.75	0.74	0.64
**Phosphodiesters**	Phosphocreatine	1.13	0.75	0.74	0.68	0.94
	Phosphoryl Choline	1.07	0.68	0.65	0.67	0.93
	O - Phosphoryl -Ethanolamine	1.08	0.68	0.75	0.70	0.91
	Methylene Diphosphonic Acid	0.84	0.37	0.49	0.45	0.53
**Phosphotriesters**	Triethyl Phosphate	0.63	0.54	0.49	0.48	0.56
**Phosphonates**	Phosphono Aceti Acid	1.02	1.20	0.77	0.76	0.84
	2 - Aminoethyl Phosphonic Acid	0.99	1.06	0.68	0.72	0.87
**Inositol Phosphates / Polyphosphates**	Inositol Hexaphosphate	0.61	0.42	0.49	0.54	0.51
**Other Organic Phosphorous compounds**	Cysteamine - S -Phosphate	0.82	0.94	0.95	0.76	0.74
	Phospho - L -Arginine	0.79	0.95	0.94	0.78	0.75

ST4d. Sulfurated Microwell Plate (Biolog® PM4A)

Functional classification	Individual Source	Absorbance*				
		NL	PuB	PoP	SL	VB
**Sulfates**		0.74	0.83	0.53	0.62	0.63
**Thiosulfates**		0.75	0.82	0.66	0.64	0.74
**Tetrathionates**		0.74	0.83	0.69	0.62	0.73
**Thiophosphates and Dithiophosphates**	Thiophosphate	0.78	0.60	0.65	0.69	0.55
	Dithiophosphate	0.81	0.61	0.67	0.69	0.55
**Sulfur-containing amino acids and derivatives**	L - Cysteine	0.87	0.87	0.65	0.64	0.69
	D - Cysteine	0.77	0.86	0.66	0.65	0.63
	L - Cysteinyl -Glycine	0.85	0.68	0.64	0.65	0.65
	L - Cysteic Acid	0.83	1.00	0.50	0.68	0.75
	Cysteamine	0.84	0.59	0.44	0.73	0.64
	L - Cysteine Sulfinic Acid	0.72	0.68	0.67	0.65	0.55

	N - Acety l - L -Cysteine	0.88	0.69	0.60	0.65	0.56
	S - Methyl - L -Cysteine	0.65	0.84	0.67	0.63	0.58
	Cystathionine	0.81	0.74	0.66	0.66	0.64
	Lanthionine	0.71	0.83	0.60	0.69	0.76
	Glutathione	0.75	0.67	0.56	0.65	0.43
	D, L - Ethionine	0.67	0.67	0.63	0.76	0.59
	L - Methionine	0.74	0.53	0.64	0.64	0.78
	D - Methionine	0.68	0.77	0.62	0.64	0.67
	Glycyl - L -Methionine	0.62	0.63	0.74	0.74	0.70
	N - Acetyl - D ,L Methionine	0.62	0.76	0.74	0.65	0.64
	L - Methionine Sulfoxide	0.69	0.66	0.58	0.71	0.66
	L - Methionine Sulfone	0.69	0.66	0.62	0.71	0.63
	L - Djenkolic Acid	0.67	0.46	0.55	0.65	0.47
	Taurocholic Acid	0.54	0.69	0.53	0.65	0.46
	Taurine	0.82	0.74	0.66	0.62	0.65
	Hypotaurine	0.60	0.84	0.67	0.54	0.66
**Other Organic Sulfur Compounds**	Thiourea	0.72	0.57	0.81	0.68	0.57
	1 -Thio - β - D -Glucose	0.56	0.59	0.58	0.64	0.29
	D, L -Lipoamide	0.73	0.40	0.73	0.66	0.55
	p- Amino Benzene Sulfonic Acid	0.67	0.67	0.71	0.64	0.43
	Butane Sulfonic Acid	0.65	0.73	0.61	0.65	0.63
	2 -Hydroxyethane Sulfonic Acid	0.71	0.70	0.69	0.58	0.67
	Methane Sulfonic Acid	0.89	0.67	0.83	0.66	0.58
	Tetramethylene Sulfone	0.53	0.41	0.69	0.73	0.36

Table S6. Primer sets and PCR conditions used for the quantification of bacterial functional genes

Target	Gen	Name	Primer Sequence (5' a 3')	qPCR Conditions	Amplicon Size	Slope / R^2 (%)/ Efficiency (%)	LOD / LOQ (copy number)	Reference
**Total Bacteria**	16S rRNA	799F	AACMGGATTAGATACCCKG	12 min at 95 °C, and 40 cycles at 95 °C per 15 sec and 60 °C per 30 sec, finalized by 30 sec at 72 °C	316	-3.354 / 99.46 / 94.3	3 / 59	Shade et al. (2013)
		1115R	AGGGTTGCGCTCGTTG					
**P**	*pho*D	ALPS-F730	CAGTGGGACGACCACGAGGT	12 min at 95 °C, and 40 cycles at 95 °C per 15 sec and 60 °C per 1 min	370	-3.437 / 99.24 / 95.4	4 / 116	Sakurai et al. (2008)
		ALPS-R1101	GAGGCCGATCGGCATGTCG					
	*pqq*C	pqqC_F	AACCGCTTCTACTACCAG	12 min at 95 °C, and 40 cycles at 95 °C per 5 sec and 60 °C per 30 sec	305	-3.253 / 99.13 / 103	4 / 142	Zheng et al. (2017)
		pqqC_R	GCGAACAGCTCGGTCAG					
**N**	*nif*H	PolF	TGCGAYCCSAARGCBGACTC	12 min at 95 °C, and 40 cycles at 95 °C per 15 sec, 55 °C per 1 min, finalized by 30 sec at 72 °C	359	-3.555 / 98.61 / 91.1	6 / 367	Poly (2001)
		PolR	ATSGCCATCATYTCRCCGGA					

	*amo*A	amoA-1F	GGGGTTTCTACTGGTGGT	12 min at 95 °C, and 40 cycles at 95 °C per 15 sec, 60 °C per 30 sec, finalized by 30 sec at 72 °C	491	-3.217 / 99.52 / 104.6	2 / 13	Rotthauwe (1997)
		amoA-2R	CCCCTCGGGAAAGCCTTCT					
	*nos*Z	nosZ2F	CGCRACGGCAASAAGGTSMSSGT		267	-3.130 / 98.85 / 108.7	3 / 51	Henry (2006)
		nosZ2R	CAKRTGCAKSGCRTGGCAGAA					
C	*chi*A	chiF2	GACGGCATCGACATCGATTGG		436	-3.607 / 99.85 / 93.2	2 / 5	Köllner et al. (2012)
		chiR	CSGTCCAGCCGCGSCCRTA					
	*mcr*A	mcrA-rev	CGTTCATBGCGTAGTTVGGRTAGT	12 min at 95 °C, and 40 cycles at 95 °C per 15 sec and 56 °C per 45 sec, finalized by 30 sec at 72 °C	271	-3.144 / 98.84 / 108	4 / 106	Cisek et al. (2023)
		mcrA_F3	CTTGAARMTCACTTCGGTGGWTC					
S	*sox*B	soxB195_710F	ATCGGYCAGGCYTTYCCSTA		512	-3.481 / 99.99 / 91.7	1 / 2	Tourna et al. (2014)
		soxB195_1184	MAVGTGCCGTTGAARTTGC					
	*dsr*A	dsr1F+	ACSCACTGGAAGCACGGCGG		251	-3.538 / 98.96 / 93.8	5 / 165	Carolan et al. (2015)
		dsrR	GTGGMRCCGTGCAKRTTGG					

Table S7. Richness and Alpha diversity of sediment samples from Lake Villarrica.

Site	Richness	Diversity Indexes		
	**Observed ASVs**	**Shannon**	**Pielou´s Evenness**	**Faith´s Phylogenetic**
**NL**	2,432 ± 161 ^{A}	10.23 ± 0.1 ^{B}	0.90 ± 0.0 ^{AB}	393.5 ± 51.4 ^{A}
**PuB**	2,226 ± 39 ^{A}	9.92 ± 0.1 ^{B}	0.89 ± 0.0 ^{B}	359.6 ± 25.0 ^{A}
**PoP**	2,133 ± 386 ^{A}	9.45 ± 0.1 ^{C}	0.85 ± 0.0 ^{C}	376.0 ± 27.2 ^{A}
**SL**	2,944 ± 601 ^{A}	10.57 ± 0.2 ^{A}	0.92 ± 0.1 ^{A}	346.6 ± 19.1 ^{A}
**VB**	2,517 ± 189 ^{A}	10.1 ± 0.1 ^{B}	0.89 ± 0.0 ^{B}	364.3 ± 25.3 ^{A}

Table S8. Spearman's rank correlation analysis between qPCR-measured and PICRUSt2-predicted gene abundances across sampling sites

PICRUSt	Gene Abundance (qPCR)	Sites									
		NL		PuB		PoP		SL		VB	
		ϱ Spearman	Prob > |ϱ|	ϱ Spearman	Prob > |ϱ|	ϱ Spearman	Prob > |ϱ|	ϱ Spearman	Prob > |ϱ|	ϱ Spearman	Prob > |ϱ|
*mcr*A (K00399)	16S rRNA	10	<.0001	10	<.0001	-10	<.0001	0.5	0.6667	0.5	0.6667
	*pho*D	10	<.0001	0.5	0.6667	10	<.0001	10	<.0001	0.5	0.6667
	*pqq*C	-0.5	0.6667	-0.5	0.6667	0.5	0.6667	0.5	0.6667	10	<.0001
	*nif*H	-0.5	0.6667	-10	<.0001	-0.5	0.6667	-10	0.6667	0	10,000
	*amo*A	-0.5	0.6667	0.5	0.6667	0.5	0.6667	-10,000	<.0001	0.5	0.6667
	*nos*Z	10	<.0001	0	10,000	-10	<.0001	0.5	0.6667	-0.5	0.6667
	*chi*A	0.5	0.6667	-0.5	0.6667	0	10,000	0.5	0.6667	0.5	0.6667
	*mcr*A	0.5	0.6667	0.5	0.6667	10	<.0001	-10	<.0001	-0.5	0.6667
	*sox*B	-10	<.0001	10	<.0001	-10	<.0001	0.5	0.6667	-10	<.0001
	*dsr*A	-0.5	0.6667	10	<.0001	-0.5	0.6667	-0.5	0.6667	-0.5	0.6667
*chi*A (K13381)	16S rRNA	.	.	.	.	.	.	.	.	0	10,000
	*pho*D	.	.	.	.	.	.	.	.	0.866	0.3333
	*pqq*C	.	.	.	.	.	.	.	.	0.866	0.3333
	*nif*H	.	.	.	.	.	.	.	.	0.5	0.6667
	*amo*A	.	.	.	.	.	.	.	.	0	10,000
	*nos*Z	.	.	.	.	.	.	.	.	0	10,000
	*chi*A	.	.	.	.	.	.	.	.	0	10,000
	*mcr*A	.	.	.	.	.	.	.	.	0	10,000
	*sox*B	.	.	.	.	.	.	.	.	-0.866	0.3333
	*dsr*A	.	.	.	.	.	.	.	.	-0.866	0.3333
*nif*H (K02588)	16S rRNA	0.5	0.6667	0.5	0.6667	-0.5	0.6667	0.5	0.6667	0.5	0.6667
	*pho*D	0.5	0.6667	-0.5	0.6667	0.5	0.6667	10	<.0001	0.5	0.6667
	*pqq*C	0.5	0.6667	-10	<.0001	-0.5	0.6667	0.5	0.6667	10	<.0001
	*nif*H	0.5	0.6667	-0.5	0.6667	0.5	0.6667	-0.5	0.6667	0	10,000
	*amo*A	0.5	0.6667	-0.5	0.6667	-0.5	0.6667	-10	<.0001	0.5	0.6667
	*nos*Z	0.5	0.6667	0.866	0.3333	-0.5	0.6667	0.5	0.6667	-0.5	0.6667
	*chi*A	-0.5	0.6667	0.5	0.6667	-0.866	0.3333	0.5	0.6667	0.5	0.6667
	*mcr*A	-0.5	0.6667	-0.5	0.6667	0.5	0.6667	-10	<.0001	-0.5	0.6667
	*sox*B	-0.5	0.6667	0.5	0.6667	-0.5	0.6667	0.5	0.6667	-10	<.0001
	*dsr*A	-10	<.0001	0.5	0.6667	0.5	0.6667	-0.5	0.6667	-0.5	0.6667
*amo*A (K10944)	16S rRNA	10	<.0001	-0.5	0.6667	-10	<.0001	0.5	0.6667	0.5	0.6667
	*pho*D	10	<.0001	-10	<.0001	10	<.0001	10	<.0001	0.5	0.6667
	*pqq*C	-0.5	0.6667	-0.5	0.6667	0.5	0.6667	0.5	0.6667	10	<.0001
	*nif*H	-0.5	0.6667	0.5	0.6667	-0.5	0.6667	-0.5	0.6667	0	10,000
	*amo*A	-0.5	0.6667	-10	<.0001	0.5	0.6667	-10	<.0001	0.5	0.6667
	*nos*Z	10	<.0001	0.866	0.3333	-10	<.0001	0.5	0.6667	-0.5	0.6667
	*chi*A	0.5	0.6667	10	<.0001	0	10,000	0.5	0.6667	0.5	0.6667

	*mcr*A	0.5	0.6667	-10	<.0001	10	<.0001	-10	<.0001	-0.5	0.6667
	*sox*B	-10	<.0001	-0.5	0.6667	-10	<.0001	0.5	0.6667	-10	<.0001
	*dsr*A	-0.5	0.6667	-0.5	0.6667	-0.5	0.6667	-0.5	0.6667	-0.5	0.6667
*nos*Z (K00376)	16S rRNA	10	<.0001	0.5	0.6667	-10	<.0001	0.5	0.6667	0.5	0.6667
	*pho*D	10	<.0001	-0.5	0.6667	10	<.0001	10	<.0001	0.5	0.6667
	*pqq*C	-0.5	0.6667	-10	<.0001	0.5	0.6667	0.5	0.6667	10	<.0001
	*nif*H	-0.5	0.6667	-0.5	0.6667	-0.5	0.6667	-0.5	0.6667	0	10,000
	*amo*A	-0.5	0.6667	-0.5	0.6667	0.5	0.6667	-10	<.0001	0.5	0.6667
	*nos*Z	10	<.0001	0.866	0.3333	-10	<.0001	0.5	0.6667	-0.5	0.6667
	*chi*A	0.5	0.6667	0.5	0.6667	0	10,000	0.5	0.6667	0.5	0.6667
	*mcr*A	0.5	0.6667	-0.5	0.6667	10	<.0001	-10	<.0001	-0.5	0.6667
	*sox*B	-10	<.0001	0.5	0.6667	-10	<.0001	0.5	0.6667	-10	<.0001
	*dsr*A	-0.5	0.6667	0.5	0.6667	-0.5	0.6667	-0.5	0.6667	-0.5	0.6667
*pqq*C (K06137)	16S rRNA	10	<.0001	0.5	0.6667	-10	<.0001	0.5	0.6667	0.5	0.6667
	*pho*D	10	<.0001	-0.5	0.6667	10	<.0001	10	<.0001	0.5	0.6667
	*pqq*C	-0.5	0.6667	-10	<.0001	0.5	0.6667	0.5	0.6667	10	<.0001
	*nif*H	-0.5	0.6667	-0.5	0.6667	-0.5	0.6667	-0.5	0.6667	0	10,000
	*amo*A	-0.5	0.6667	-0.5	0.6667	0.5	0.6667	-10	<.0001	0.5	0.6667
	*nos*Z	10	<.0001	0.866	0.3333	-10	<.0001	0.5	0.6667	-0.5	0.6667
	*chi*A	0.5	0.6667	0.5	0.6667	0	10,000	0.5	0.6667	0.5	0.6667
	*mcr*A	0.5	0.6667	-0.5	0.6667	10	<.0001	-10	<.0001	-0.5	0.6667
	*sox*B	-10	<.0001	0.5	0.6667	-10	<.0001	0.5	0.6667	-10	<.0001
	*dsr*A	-0.5	0.6667	0.5	0.6667	-0.5	0.6667	-0.5	0.6667	-0.5	0.6667
*pho*D (K01113)	16S rRNA	0.5	0.6667	10	<.0001	-0.5	0.6667	0.5	0.6667	10	<.0001
	*pho*D	0.5	0.6667	0.5	0.6667	0.5	0.6667	10	<.0001	-0.5	0.6667
	*pqq*C	0.5	0.6667	-0.5	0.6667	-0.5	0.6667	0.5	0.6667	0.5	0.6667
	*nif*H	0.5	0.6667	-10	<.0001	0.5	0.6667	-0.5	0.6667	-0.866	0.3333
	*amo*A	0.5	0.6667	0.5	0.6667	-0.5	0.6667	-10	<.0001	10	<.0001
	*nos*Z	0.5	0.6667	0	10,000	-0.5	0.6667	0.5	0.6667	-10	<.0001
	*chi*A	-0.5	0.6667	-0.5	0.6667	-0.866	0.3333	0.5	0.6667	10	<.0001
	*mcr*A	-0.5	0.6667	0.5	0.6667	0.5	0.6667	-10	<.0001	-10	<.0001
	*sox*B	-0.5	0.6667	10	<.0001	-0.5	0.6667	0.5	0.6667	-0.5	0.6667
	*dsr*A	-10	<.0001	10	<.0001	0.5	0.6667	-0.5	0.6667	0.5	0.6667
*dsr*A (K11180)	16S rRNA	0.5	0.6667	10	<.0001	-10	<.0001	0.5	0.6667	0.5	0.6667
	*pho*D	0.5	0.6667	0.5	0.6667	10	<.0001	10	<.0001	-10	<.0001
	*pqq*C	0.5	0.6667	-0.5	0.6667	0.5	0.6667	0.5	0.6667	-0.5	0.6667
	*nif*H	0.5	0.6667	-10	<.0001	-0.5	0.6667	-0.5	0.6667	-0.866	0.3333
	*amo*A	0.5	0.6667	0.5	0.6667	0.5	0.6667	-10	<.0001	0.5	0.6667
	*nos*Z	0.5	0.6667	0	10,000	-10	<.0001	0.5	0.6667	-0.5	0.6667
	*chi*A	-0.5	0.6667	-0.5	0.6667	0	10,000	0.5	0.6667	0.5	0.6667
	*mcr*A	-0.5	0.6667	0.5	0.6667	10	<.0001	-10	<.0001	-0.5	0.6667
	*sox*B	-0.5	0.6667	10	<.0001	-10	<.0001	0.5	0.6667	0.5	0.6667
	*dsr*A	-10	<.0001	10	<.0001	-0.5	0.6667	-0.5	0.6667	10	<.0001
	16S rRNA	0.5	0.6667	0.5	0.6667	-0.5	0.6667	0.5	0.6667	0.5	0.6667

*sox*B (K17244)	*pho*D	0.5	0.6667	-0.5	0.6667	0.5	0.6667	10	<.0001	0.5	0.6667
	*pqq*C	0.5	0.6667	-10	<.0001	-0.5	0.6667	0.5	0.6667	10	<.0001
	*nif*H	0.5	0.6667	-0.5	0.6667	0.5	0.6667	-0.5	0.6667	0	10,000
	*amo*A	0.5	0.6667	-0.5	0.6667	-0.5	0.6667	-10	<.0001	0.5	0.6667
	*nos*Z	0.5	0.6667	0.866	0.3333	-0.5	0.6667	0.5	0.6667	-0.5	0.6667
	*chi*A	-0.5	0.6667	0.5	0.6667	-0.866	0.3333	0.5	0.6667	0.5	0.6667
	*mcr*A	-0.5	0.6667	-0.5	0.6667	0.5	0.6667	-10	<.0001	-0.5	0.6667
	*sox*B	-0.5	0.6667	0.5	0.6667	-0.5	0.6667	0.5	0.6667	-10	<.0001
	*dsr*A	-10	<.0001	0.5	0.6667	0.5	0.6667	-0.5	0.6667	-0.5	0.6667

Spearman's rank correlation (ρ) and corresponding p-values between qPCR-measured and PICRUSt2-predicted gene abundances across sampling sites (n = 5). Correlations with $p < 0.05$ were interpreted as significant.

Table S9. Results of Inertia, proportion constrained, r^2, adjusted r^2, and *P*-value of each variable included in the partial redundancy analysis (RDA) of this study.

Variable	Inertia	Proportion Constrained	R^2 (Proportion Total)	Adjusted R^2 (Sequential)	P-value
***pho*D**	0.082357	0.4338	0.421	0.3764	0.001 ***
***mcr*A**	0.042039	0.2215	0.2149	0.5752	0.001 ***
***nos*Z**	0.027223	0.1434	0.1391	0.7136	0.001 ***
**TS**	0.021431	0.1129	0.1095	0.8384	0.001 ***
***chi*A**	0.013152	0.0693	0.0672	0.925	0.001 ***
***amo*A**	0.003633	0.0191	0.0186	0.9481	0.012 *
***pqq*C, *nif*H, *sox*B, *dsr*A, OM, TN, TP, TC**	0.000001				n.s
Total Constrained	0.189836	1			

Proportion Constrained is the proportion of Inertia from the constrained Inertia.
r^2 is the proportion of Inertia from the total Inertia.
P-value is the significance of that partial RDA model tested by the permutation test.

Figure S1. Standardized (z-score) plots illustrating the nutrient gradient among sample sites

a)

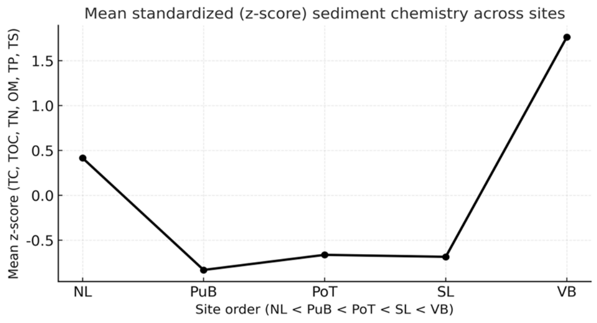

b)

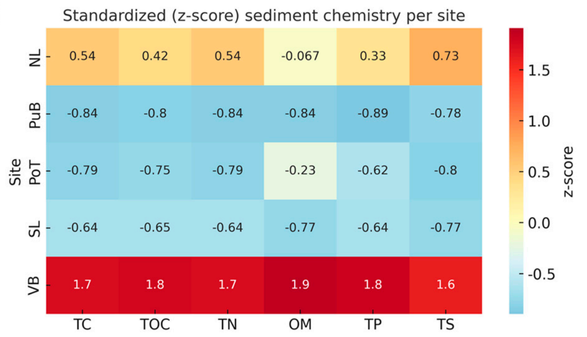

To verify the expected a priori spatial gradient (NL < PuB < PoT < SL < VB) using sediment chemistry variables (TC, TOC, TN, OM, TP, TS) were standardized to z-scores. Then, **a)** line plot showing the mean z-score per site confirms a monotonic increase toward Villarrica Bay (VB), whereas **b)** a heatmap of standardized values highlights consistent enrichment of TOC, TN, TP, and TS at VB.

Figure S2. Substrate layout and functional category mapping for Biolog® **a)** EcoPlates™ with 31 C sources plus controls, **b)** PM3B with 95 N sources plus controls, and **c)** PM4A with 59 P sources and 35 S sources plus controls.

a)

	1	2	3	4	5	6	7	8	9	10	11	12
A	Control	β-Methyl-D-Glucoside	D-Galactonic Acid γ-Lactone	L-Arginine	Control	β-Methyl-D-Glucoside	D-Galactonic Acid γ-Lactone	L-Arginine	Control	β-Methyl-D-Glucoside	D-Galactonic Acid γ-Lactone	L-Arginine
B	Pyruvic Acid Methyl Ester	D-Xylose	D-Galacturonic Acid	L-Asparagine	Pyruvic Acid Methyl Ester	D-Xylose	D-Galacturonic Acid	L-Asparagine	Pyruvic Acid Methyl Ester	D-Xylose	D-Galacturonic Acid	L-Asparagine
C	Tween 40	i-Erythritol	2-Hydroxy Benzoic Acid	L-Phenylalanine	Tween 40	i-Erythritol	2-Hydroxy Benzoic Acid	L-Phenylalanine	Tween 40	i-Erythritol	2-Hydroxy Benzoic Acid	L-Phenylalanine
D	Tween 80	D-Mannitol	4-Hydroxy Benzoic Acid	L-Serine	Tween 80	D-Mannitol	4-Hydroxy Benzoic Acid	L-Serine	Tween 80	D-Mannitol	4-Hydroxy Benzoic Acid	L-Serine
E	α-Cyclodextrin	N-Acetyl-D-Glucosamine	γ-Hydroxybutyric Acid	L-Threonine	α-Cyclodextrin	N-Acetyl-D-Glucosamine	γ-Hydroxybutyric Acid	L-Threonine	α-Cyclodextrin	N-Acetyl-D-Glucosamine	γ-Hydroxybutyric Acid	L-Threonine
F	Glycogen	D-Glucosaminic Acid	Itaconic Acid	b-Glycyl-L-Glutamic Acid	Glycogen	D-Glucosaminic Acid	Itaconic Acid	b-Glycyl-L-Glutamic Acid	Glycogen	D-Glucosaminic Acid	Itaconic Acid	b-Glycyl-L-Glutamic Acid
G	D-Cellobiose	Glucose-1-Phosphate	α-Ketobutyric Acid	Phenylethyl-amine	D-Cellobiose	Glucose-1-Phosphate	α-Ketobutyric Acid	Phenylethyl-amine	D-Cellobiose	Glucose-1-Phosphate	α-Ketobutyric Acid	Phenylethyl-amine
H	α-D-Lactose	D,L-α-Glycerol Phosphate	D-Malic Acid	Putrescine	α-D-Lactose	D,L-α-Glycerol Phosphate	D-Malic Acid	Putrescine	α-D-Lactose	D,L-α-Glycerol Phosphate	D-Malic Acid	Putrescine

Carbon sources — Controls, Carboxilic acids, Glucides, Amino acids, Amines, Phenolic compounds, Polymers

b)

	1	2	3	4	5	6	7	8	9	10	11	12
A	Control	Ammonia	Nitrite	Nitrate	Urea	Biuret	L-Alanine	L-Arginine	L-Asparagine	L-Aspartic Acid	L-Cysteine	L-Glutamic Acid
B	L-Glutamine	Glycine	L-Histidine	L-Isoleucine	L-Leucine	L-Lysine	L-Methionine	L-Phenylalanine	L-Proline	L-Serine	L-Threonine	L-Tryptophan
C	L-Tyrosine	L-Valine	D-Alanine	D-Asparagine	D-Aspartic Acid	D-Glutamic Acid	D-Lysine	D-Serine	D-Valine	L-Citrulline	L-Homoserine	L-Ornithine
D	N-Acetyl-L-Glutamic Acid	N-Phthaloyl-L-Glutamic Acid	L-Pyroglutamic Acid	Hydroxylamine	Methylamine	N-Amylamine	N-Butylamine	Ethylamine	Ethanolamine	Ethylenediamine	Putrescine	Agmatine
E	Histamine	β-Phenylethyl-amine	Tyramine	Acetamide	Formamide	Glucuronamide	D,L-Lactamide	D-Glucosamine	D-Galactosamine	D-Mannosamine	N-Acetyl-D-Glucosamine	N-Acetyl-D-Galactosamine
F	N-Acetyl-D-Mannosamine	Adenine	Adenosine	Cytidine	Cytosine	Guanine	Guanosine	Thymine	Thymidine	Uracil	Uridine	Inosine
G	Xanthine	Xanthosine	Uric Acid	Alloxan	Allantoin	Parabanic Acid	D,L-α-Amino-N-Butyric Acid	γ-Amino-N-Butyric Acid	ε-Amino-N-Caproic Acid	D,L-α-Amino-Caprylic Acid	δ-Amino-N-Valeric Acid	α-Amino-N-Valeric Acid
H	Ala-Asp	Ala-Gln	Ala-Glu	Ala-Gly	Ala-His	Ala-Leu	Ala-Thr	Gly-Asn	Gly-Gln	Gly-Glu	Gly-Met	Met-Ala

Nitrogen sources

- Control
- Ammonia
- Nitrite
- Nitrate
- Amino acids and derivates
- Amines and derivates
- Amides and derivates
- Nucleobases and derivates
- Peptides

c)

	1	2	3	4	5	6	7	8	9	10	11	12
A	Control	Phosphate	Pyrophosphate	Trimeta-phosphate	Tripoly-phosphate	Triethyl Phosphate	Hypophosphite	Adenosine- 2′-monophosphate	Adenosine- 3′-monophosphate	Adenosine- 5′-monophosphate	Adenosine- 2′,3′-cyclic monophosphate	Adenosine- 3′,5′-cyclic monophosphate
B	Thiophosphate	Dithiophosphate	D,L-α-Glycerol Phosphate	β-Glycerol Phosphate	Carbamyl Phosphate	D-2-Phospho-Glyceric Acid	D-3-Phospho-Glyceric Acid	Guanosine- 2′-monophosphate	Guanosine- 3′-monophosphate	Guanosine- 5′-monophosphate	Guanosine- 2′,3′-cyclic monophosphate	Guanosine- 3′,5′-cyclic monophosphate
C	Phosphoenol Pyruvate	Phospho-Glycolic Acid	D-Glucose-1-Phosphate	D-Glucose-6-Phosphate	2-Deoxy-D-Glucose 6-Phosphate	D-Glucosamine-6-Phosphate	6-Phospho-Gluconic Acid	Cytidine- 2′-monophosphate	Cytidine- 3′-monophosphate	Cytidine- 5′-monophosphate	Cytidine- 2′,3′-cyclic monophosphate	Cytidine- 3′,5′-cyclic monophosphate
D	D-Mannose-1-Phosphate	D-Mannose-6-Phosphate	Cysteamine-S-Phosphate	Phospho-L-Arginine	O-Phospho-D-Serine	O-Phospho-L-Serine	O-Phospho-L-Threonine	Uridine- 2′-monophosphate	Uridine- 3′-monophosphate	Uridine- 5′-monophosphate	Uridine- 2′,3′-cyclic monophosphate	Uridine- 3′,5′-cyclic monophosphate
E	O-Phospho-D-Tyrosine	O-Phospho-L-Tyrosine	Phosphocreatine	Phosphoryl Choline	O-Phosphoryl-Ethanolamine	Phosphono Acetic Acid	2-Aminoethyl Phosphonic Acid	Methylene Diphosphonic Acid	Thymidine- 3′-monophosphate	Thymidine- 5′-monophosphate	Inositol Hexaphosphate	Thymidine 3′,5′-cyclic monophosphate
F	Control	Sulfate	Thiosulfate	Tetrathionate	Thiophosphate	Dithiophosphate	L-Cysteine	D-Cysteine	L-Cysteinyl-Glycin	L-Cysteic Acid	Cysteamine	L-Cysteine Sulfinic Acid
G	N-Acetyl-L-Cysteine	S-Methyl-L-Cysteine	Cystathionine	Lanthionine	Glutathione	D,L-Ethionine	L-Methionine	D-Methionine	Glycyl-L-Methionine	N-Acetyl-D,L-Methionine	L- Methionine Sulfoxide	L-Methionine Sulfone
H	L-Djenkolic Acid	Thiourea	1-Thio-β-D-Glucose	D,L-Lipoamide	Taurocholic Acid	Taurine	Hypotaurine	p-Amino Benzene Sulfonic Acid	Butane Sulfonic Acid	2-Hydroxyethane Sulfonic Acid	Methane Sulfonic Acid	Tetramethylene Sulfone

Phosphorus sources (lines A to E)

- Control
- Phosphate
- Polyphosphates
- Phosphonates
- Phosphomonoesters
- Phosphodiesters
- Phosphotriesters
- Inositol Phosphate
- Other P_0 compounds
- Hypo and Thiophosphates

Sulfur sources (lines F to H)

- Control
- Sulfates
- Thiosulfates
- Tetrathionates
- Thio and Dithiophasphates
- S-amino acids and derivates
- Other S_0 compounds

Figure S3. Beta diversity

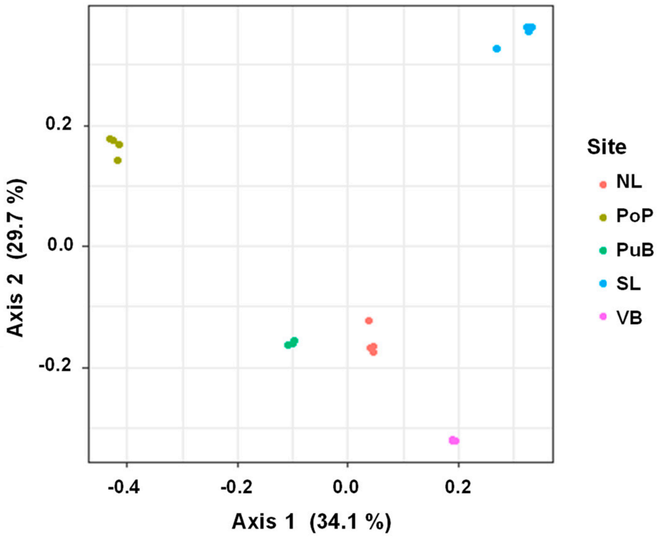

Principal Coordinate Analysis (PCoA) based on Bray–Curtis dissimilarities among sediment bacterial communities from the five sampling sites (NL, PuB, PoP, SL, and VB). Each point represents one sediment sample, colored by site. The percentage of variation explained by each axis corresponds to its eigenvalue, which quantifies the proportion of total community compositional variance captured by that axis. Axes 1 and 2 explain 34.1% and 29.7% of the variance, respectively, illustrating the gradient from low- to high-nutrient sites.

Figure S4. NSTI (Nearest Sequenced Taxon Index)

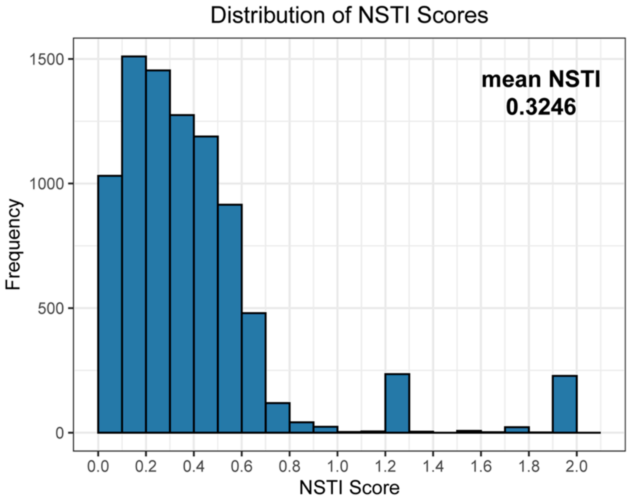

NSTI values indicate phylogenetic distance to reference genomes; the mean score (0.3246) falls within the expected range for sediment microbiomes, supporting cautious interpretation of predicted functions.
